# Autophagy Dysregulation in ALS: When Protein Aggregates Get Out of Hand

**DOI:** 10.3389/fnmol.2017.00263

**Published:** 2017-08-22

**Authors:** Nandini Ramesh, Udai Bhan Pandey

**Affiliations:** ^1^Department of Human Genetics, University of Pittsburgh Graduate School of Public Health Pittsburgh, PA, United States; ^2^Division of Child Neurology, Department of Pediatrics, Children’s Hospital of Pittsburgh, University of Pittsburgh Medical Center Pittsburgh, PA, United States; ^3^Department of Neurology, University of Pittsburgh School of Medicine Pittsburgh, PA, United States

**Keywords:** ALS, autophagy, SOD1, TDP-43, FUS, C9orf72, neurodegeneration, motor neuron disease

## Abstract

Amyotrophic lateral sclerosis (ALS) is a neurodegenerative disorder that results from the loss of upper and lower motor neurons. One of the key pathological hallmarks in diseased neurons is the mislocalization of disease-associated proteins and the formation of cytoplasmic aggregates of these proteins and their interactors due to defective protein quality control. This apparent imbalance in the cellular protein homeostasis could be a crucial factor in causing motor neuron death in the later stages of the disease in patients. Autophagy is a major protein degradation pathway that is involved in the clearance of protein aggregates and damaged organelles. Abnormalities in autophagy have been observed in numerous neurodegenerative disorders, including ALS. In this review, we discuss the contribution of autophagy dysfunction in various *in vitro* and *in vivo* models of ALS. Furthermore, we examine the crosstalk between autophagy and other cellular stresses implicated in ALS pathogenesis and the therapeutic implications of regulating autophagy in ALS.

## Introduction

Amyotrophic Lateral Sclerosis (ALS) is a fatal, age-related neurodegenerative disorder characterized by progressive loss of both upper and lower motor neurons in the brain and spinal cord (Rowland and Shneider, 2001; Pasinelli and Brown, 2006). ALS is the most common motor neuron disorder with an incidence rate of 2.7 per 100,000 Caucasians, and typically has an age-of-onset varying between 50–65 years with a median age of onset at 64 years (Zarei et al., 2015). The progressive degeneration of motor neurons is followed by muscle atrophy, spasticity and quadriplegia, culminating in death within 3–5 years of disease onset due to respiratory failure (Rowland and Shneider, 2001). Current treatments include Riluzole, which extends survival by 2–3 months in a subset of patients, and the recently FDA-approved drug, Radicava, which slows down the decline of physical function. However, there are currently no cures for ALS (Traynor et al., 2003; Yoshino and Kimura, 2006; Abe et al., 2014; Ittner et al., 2015).

ALS is mainly categorized into two forms: familial ALS (fALS) and sporadic ALS (sALS). SALS is more common, comprising 90%–95% of cases and does not have any known genetic inheritance. On the other hand, fALS affects 5%–10% of cases and is hereditary (Zarei et al., 2015). Early linkage studies in fALS families led to discovery of mutations in superoxide dismutase 1 (mSOD1), thus establishing the first genetic link to ALS (Turner et al., 2013). With the advent of whole-exome sequencing, there has been a tremendous increase in discovery of new disease-causing genes in ALS, notably fused in sarcoma (FUS), TAR DNA-binding protein 43 (TDP-43), *C9orf72*, *optineurin (OPTN)*, *sequestosome 1 (SQSTM1)*, *ubiquilin 2 (UBQLN2)*, *dynactin (DCTN1)*, *MATR3* and *valosin-containing protein* (*VCP*; Renton et al., 2014).

Like many other neurodegenerative disorders, a key pathological hallmark of ALS is the mislocalization of proteins and presence of cytoplasmic aggregates in motor neurons and surrounding cells, suggesting defects in the machinery that regulates protein homeostasis. Cells have two major protein degradation pathways: the ubiquitin proteasome system (UPS) and the autophagy-lysosome pathway. The UPS, the major proteolytic pathway in the cell, degrades short-lived, soluble proteins. Autophagy, on the other hand, is responsible for degrading relatively long-lived, cytoplasmic proteins, soluble and insoluble misfolded proteins, and also entire organelles. In this review, we discuss the importance of the autophagy pathway in the context of ALS. In addition, we discuss the interaction of autophagy with other biological pathways relevant to motor neuron degeneration and provide perspective on the role of autophagy in the overall pathogenesis of ALS.

## Dysfunction in Protein Homeostasis: Evidence from ALS Pathology

Proteins are dynamic molecules within a cell, undergoing synthesis, proper folding and assembly and timely degradation. The balance between these events is maintained by a network of pathways called the proteostatic network (Balch et al., 2008). This network is maintained by various factors that include molecular chaperones, their regulators, and proteolytic machinery (Crippa et al., 2010; Carra et al., 2012). In addition to maintaining protein levels within the proteome, proteolysis also plays a crucial role in the elimination of dysfunctional proteins such as misfolded proteins and aggregates (Balch et al., 2008).

A crucial pathological feature of ALS includes accumulation of insoluble protein aggregates in degenerating motor neurons and surrounding oligodendrocytes in the spinal cord, hippocampus, cerebellum and frontal and temporal cortices (Balch et al., 2008). These aggregates are typically formed by misfolded proteins that have lost their native conformation. Formation of misfolded protein aggregates is a normal physiological phenomenon; the cell continuously employs quality control mechanisms to either degrade the misfolded proteins to avoid aggregate formation or clears aggregates that have already formed. However, the persistence of these aggregates in diseased neurons suggests a disruption in the mechanisms normally responsible for misfolded protein turnover and aggregate clearance. Pathological aggregates may stimulate aberrant cellular interactions between proteins or disrupt cellular function by sequestering proteins within aggregates, thus contributing to cytotoxicity.

The discovery of disease-causing mutations in genes, particularly in *SOD1, TDP-43, FUS, UBQLN2, OPTN, SQSTM1* and *C9orf72*, has enabled pathophysiologists to identify and characterize the corresponding proteins from patient tissues (Balch et al., 2008). In both familial and sporadic cases of ALS, immunofluorescence studies of post-mortem brain and spinal cord tissues have shown the presence of these proteins within aggregates (Table 1).

**Table 1:** Overview of characteristics of protein aggregates formed by SOD1, TDP-43, FUS, OPTN, UBQLN2 and C9orf72 DPR in familial and sporadic cases of ALS.

Type	Tissues affected	Characteristic pathologies	References
**SOD1**			
SOD1-fALS	Spinal cord	Lewy body-like, Fibrillized, Ubiquitinated	Shibata et al. (1996b), Bruijn et al. (1998), Kato et al. (2000) and Ross and Poirier (2004)
sALS	Spinal cord	Lewy body-like, Fibrillized, Ubiquitinated	Shibata et al. (1996a,b), Watanabe et al. (2001) and Forsberg et al. (2010)
**TDP-43**			
TDP-43-fALS	Spinal cord, Frontal cortex	Skein-like, Ubiquitinated	Giordana et al. (2010)
Sporadic ALS	Spinal cord, Hippocampus, Neocortex, Glia	Skein-like, Ubiquitinated, Hyper-phosphorylated, C-terminal fragments	Arai et al. (2006), Neumann et al. (2006), Mackenzie et al. (2007) and Giordana et al. (2010)
fALS (non-SOD1)	Spinal cord, Glia	Skein-like, Ubiquitinated	Mackenzie et al. (2007) and Tan et al. (2007)
**FUS**			
FUS-fALS	Oligodendrocytes, Spinal cord, Motor cortex, Hippocampus	Basophilic, Tangle-like, Ubiquitinated	Mackenzie et al. (2011)
sALS	Spinal cord, Glia	Skein-like, Basophilic, Ubiquitinated	Bäumer et al. (2010), Deng et al. (2010) and Mackenzie et al. (2011)
fALS (non-SOD1)	Spinal cord, Hippocampus	Skein-like	Bäumer et al. (2010), Deng et al. (2010) and Huang et al. (2011)
**OPTN**			
OPTN-fALS	Spinal cord	Ubiquitinated	Maruyama et al. (2010)
sALS	Spinal cord, Frontal cortex	Skein-like, Ubiquitinated	Maruyama et al. (2010) and Hortobágyi et al. (2011)
fALS (non-SOD1)	Spinal cord	Skein-like	Deng et al. (2011a)
sALS (SOD1)	Spinal cord	Lewy body-like, Ubiquitinated	Maruyama et al. (2010) and Deng et al. (2011a)
**UBQLN2**			
UBQLN2-fALS	Spinal cord	Skein-like, Ubiquitinated	Williams et al. (2012)
sALS	Spinal cord	Skein-like, Ubiquitinated	Deng et al. (2011b) and Williams et al. (2012)
fALS (non-SOD1)	Spinal cord	Skein-like, Ubiquitinated	Deng et al. (2011b) and Williams et al. (2012)
fALS (C9orf72)	Hippocampus, Cerebellum, Spinal cord	Skein-like, Ubiquitinated	Al-Sarraj et al. (2011), Ash et al. (2013) and Mori et al. (2013)
**C9orf72 DPR**			
fALS-C9orf72	Hippocampus, Cerebellum, Neocortex	Ubiquitinated	Mackenzie et al. (2013)

*SQSTM1/p62 protein co-localizes with aggregates in almost all familial and sporadic ALS cases.

The first evidence of aggregates was described in spinal cords of fALS patients carrying a mutation in the *SOD1* gene (Rosen et al., 1993; Bruijn et al., 1998). These SOD1-positive aggregates are sometimes polyubiquitinated and fibrillized, and are hypothesized to seed aggregation of surrounding proteins (Basso et al., 2006). Evidence of SOD1 aggregation has also been reported in post-mortem samples of spinal cords from sALS patients (Shibata et al., 1994, 1996a,b; Watanabe et al., 2001; Forsberg et al., 2010). Neurofilament aggregates containing SOD1 have also been detected in cultured motor neurons that were differentiated from induced pluripotent stem cells (iPSCs) derived from patients carrying a *SOD1* mutation (Chen et al., 2015). Thus far, SOD1 aggregates have only been observed in fALS cases containing *SOD1* mutations.

Examination of SOD1-negative fALS and sALS patients identified TDP-43 as a major component of ubiquitinated inclusions in spinal cords, hippocampus, frontal cortex neurons, and glial cells (Arai et al., 2006; Neumann et al., 2006). TDP-43 inclusions are found in motor cortices and spinal cords of nearly 97% of fALS and sALS patients. They are associated with many other neurodegenerative disorders as well, collectively termed TDP-43 proteinopathies (Sreedharan et al., 2008; Qin et al., 2014). TDP-43 proteinopathy aggregates commonly contain TDP-35 and TDP-25 species that are cleaved forms of full-length TDP-43 that are thought to be pathogenic (Arai et al., 2006; Neumann et al., 2006). In addition to accumulation of wild-type TDP-43 in SOD1-negative ALS patients, ALS-causing mutations in TDP-43 result in cytoplasmic accumulation of insoluble TDP-43 in patient neurons (Van Deerlin et al., 2008).

Similar to TDP-43, even before the discovery of pathological mutations, FUS was found to be a major protein aggregate in affected neurons in Huntington’s Disease (Zoghbi and Orr, 2000; Doi et al., 2008). In post-mortem tissues of FUS mutation carriers, FUS was shown to be enriched in cytoplasmic inclusions within the motor neuron and glial cells (Kwiatkowski et al., 2009; Vance et al., 2009). One of the unique features of FUS mutations is the vast heterogeneity in the age-of-onset, where the P525L mutation associates with relatively early onset resulting in an aggressive and juvenile form of ALS (Mackenzie et al., 2011). In the juvenile cases, FUS pathology is slightly different—FUS aggregates appear to have a filamentous structure that are associated with smaller granules (Bäumer et al., 2010; Huang et al., 2011). In addition to mutation-driven cytoplasmic inclusions, FUS-positive inclusions have also been observed in sALS cases and non-SOD1 fALS cases (Deng et al., 2010).

The most common genetic cause of ALS stems from an expansion mutation in *C9orf72* (chromosome 9 open reading frame 72), characterized by a hexanucleotide repeat (HRE) expansion of GGGGCC in the first intron of the gene (DeJesus-Hernandez et al., 2011; Renton et al., 2011). The inclusions that were first isolated post-mortem from neurons in the pyramidal, frontal and temporal cortices as well as the hippocampus were all TDP-43 immunopositive (Mackenzie et al., 2014). Further examination of inclusions from the cerebellum and pyramidal neurons of the hippocampus and neocortex revealed other aggregates that were TDP-43-negative (Mackenzie et al., 2014). Furthermore, these inclusions also contained dipeptide repeat (DPR) proteins resulting from non-ATG-initiated translation of intronic repeats (Mackenzie et al., 2013).

The discovery of ALS-associated mutations in genes encoding for proteins involved in protein degradation pathways provided compelling evidence towards a model of ALS as a disease of protein homeostatic dysregulation. These genes included *UBQLN2*, *OPTN*, *VCP* and *SQSTM1*. In particular, SQSTM1/p62 is found in almost all pathological inclusions (Majcher et al., 2015). OPTN was first detected in skein-like inclusions in spinal cords of non-SOD1 fALS and sALS patients (Deng et al., 2011a). However, studies of OPTN immunoreactivity report variable results, with OPTN observed in some inclusions and not others from SOD1 fALS and FUS fALS cases (Maruyama et al., 2010; Deng et al., 2011a; Hortobágyi et al., 2011). These discrepancies suggest a need for further studies to determine the pathology of OPTN in ALS. UBQLN2-immunopositive inclusions have been detected in spinal cords of both sALS and fALS patients with mutations in *SOD1*, *FUS*, *TDP-43* or *C9orf72* (Deng et al., 2011b; Williams et al., 2012). Interestingly, spinal cord analyses of *UBQLN2* mutation carriers revealed aggregates that are also immunopositive for other ALS-causing proteins such as FUS, OPTN and TDP-43 (Williams et al., 2012). The presence of proteasome-associated proteins within pathological aggregates indicates a cellular response to degrade the aggregates. Thus, the persistence of aggregates coupled with evidence of ALS-causing mutations in genes associated with proteasome function strongly suggests a defect in proteolysis in ALS patients.

## Autophagy

Autophagy, from the root words for “auto” = self and “phagy” = eating, is an intracellular catabolic process involved in the turnover of cellular components and nutrients such as amino acids, lipids and other metabolites to maintain cellular homeostasis (Eskelinen and Saftig, 2009). Autophagy as a cellular protein degradation mechanism first came to light when a scientist named Christian De Duve discovered a novel organelle that he termed the “lysosome” (De duve et al., 1955). It was only after the discovery of starvation-induced autophagy in yeast and autophagy-related genes (ATGs) that the mechanism itself came to prominence (Ohsumi, 2014). Autophagy maintains cellular and protein homeostasis in a tightly regulated fashion, either constitutively active in cells and/or in response to nutrient depletion or organelle damage (Shintani and Klionsky, 2004). Autophagy can be divided into three types depending on the substrate and pre-lysosomal steps involved: microautophagy, macroautophagy, and chaperone-mediated autophagy (CMA). Macroautophagy, hereby referred to as autophagy, is the only pathway through which large protein aggregates and damaged organelles are degraded (Glick et al., 2010).

### Mechanism of Autophagy

The process of autophagy begins with the formation of an autophagosome that sequesters degradation targets including soluble proteins, insoluble protein aggregates, and organelles, and delivers them to lysosomes for degradation (Ravikumar et al., 2009). This is orchestrated by a complex network of proteins encoded by ATGs (Reggiori and Klionsky, 2002; Ravikumar et al., 2009). The mechanism of autophagy can be broken down into three major steps: initiation, phagophore elongation, and autophagosome maturation/lysosomal degradation.

#### Initiation

The mammalian target of rapamycin (mTOR) is a well-characterized major regulator of autophagy initiation (Jung et al., 2010). Typically, under nutrient-rich conditions, mTOR is a negative regulator of autophagy through inactivation of ULK1 (unc51-like autophagy activating kinase 1), ATG13, and FIP200 (focal adhesion kinase family–interacting protein of 200kD). Together, these three proteins form the autophagy initiation complex (Hurley and Young, 2017). During nutrient starvation or rapamycin treatment, mTOR is released from ULK1, allowing it to form the initiation complex. The autophagy initiation complex acts in conjunction with the PI3K complex, which consists of Vps34, Beclin-1 (BECN1) and p150. These two complexes regulate the formation of a double-membraned cistern that is a precursor to the autophagosome called a phagophore (Hurley and Young, 2017).

#### Phagophore Elongation

Phagophore elongation and autophagosome formation is facilitated by two ubiquitin-like conjugation systems: the ATG12:ATG5:ATG16L system and PE-LC3 system. The first system facilitates conjugation of LC3 (light chain 3) with PE (phosphatidylethanolamine; Rubinsztein et al., 2012). The cytoplasmic form of LC3, called LC3-I, becomes lipidated by PE, which allows it to be recruited to the phagophore (Tanida et al., 2008). Once the phagophore elongates, it seals around the cytoplasmic substrates to form a double-membraned autophagosome (Rubinsztein et al., 2012). Next, the ATG12:ATG5:ATG16L complex dissociates from the membrane, leaving a part of LC3 behind, called LC3-II. Thus LC3-II is a convenient marker for autophagosome detection (Tanida et al., 2008).

#### Autophagosome Maturation/Lysosomal Degradation

The autophagosomes containing cargo destined for degradation are transported along microtubules via dynein-dynactin (DCTN1) complexes to endosomes and lysosomes (Cheng X.-T. et al., 2015). The autophagosomes fuse with endosomes to form amphisomes or with lysosomes to form autolysosomes. Amphisomes also subsequently fuse with lysosomes to form autolysosomes (Klionsky et al., 2014). These fusion processes require non-ATG protein complexes that includes Rab proteins and ATPases. In the final step, lysosomal enzymes degrade the contents within the autolysosomes (Dunn, 1994).

### Autophagy in Neurons

Adult neurons are highly specialized, post-mitotic cells that do not undergo cell division. Hence, unlike most other cell types where toxic components are diluted by cell division, neurons are susceptible to accumulation of misfolded protein aggregates and damaged organelles throughout their lifespan. They require active protein quality control processes to maintain cell viability and homeostasis (Damme et al., 2015; Maday, 2016). Neurons consist of a cell body (soma), dendrites, and axons arranged into an elongated shape. Autophagosomes form anywhere within a neuron where there are toxic substrates that need to be degraded, whereas the majority of lysosomes are located within the soma (Maday, 2016). Thus, neurons have active trafficking machinery that transport autophagosomes from the distal ends of the neurons to the soma through microtubule-associated dynein motor proteins (Cheng X.-T. et al., 2015; Maday, 2016).

The role of basal autophagy in neurons has been demonstrated in mice where deficiency of key autophagy proteins, Atg5 and Atg7, led to development of neurodegenerative and behavioral deficits (Hara et al., 2006; Komatsu et al., 2006). In addition to defects in autophagy induction, impaired autophagosome clearance has also been implicated in the pathogenesis of neurodegenerative disorders (Boland et al., 2008). Autophagy dysregulation has been implicated in various neurodegenerative disorders, especially in ones that present during adulthood such as Alzheimer’s disease, Parkinson’s disease, Multiple Sclerosis and Huntington’s disease (Kesidou et al., 2013; Menzies et al., 2015; Nah et al., 2015).

In ALS, multiple studies of post-mortem patient tissues provide evidence for autophagy dysregulation in motor neurons. A study investigating the lumbar spinal cords of patients with sALS described degenerated motor neurons with inclusions that were immunopositive for p62 and LC3-II (Sasaki, 2011). These inclusions were associated with autophagosomes and/or autolysosomes, suggesting autophagic activity in the spinal cord motor neurons of these patients (Sasaki, 2011). In addition to human post-mortem tissues, p62-positive inclusions are also significantly enriched in iPSC-derived neurons from patients with expanded GGGGCC repeats in the *C9orf72* gene (Almeida et al., 2013). In response to autophagy inhibition, these neurons were more susceptible to cell death, suggesting that autophagy function was compromised to begin with Almeida et al. (2013). Furthermore, a significant reduction in autophagy was observed in iPSC-derived neurons from ALS/frontotemporal dementia (FTLD) patients exhibiting *C9orf72* haploinsufficiency (Webster et al., 2016). Increased numbers of *in vitro* and *in vivo* models are revealing perturbations to the autophagy machinery in the pathogenesis of the ALS.

## Role of Autophagy in ALS: Lessons from *In Vitro* and *In Vivo* Models

Autophagy is a crucial mechanism that aids in the maintenance of cellular homeostasis, especially in neurons. Failure in autophagy machinery could be a significant contributor to the pathological formation of toxic aggregates in ALS. A defect in autophagy could arise from either one or more events: (i) failure in autophagy initiation; (ii) failure in autophagosome formation and/or maturation; (iii) defects in the transport of cargo within autophagosomes; (iv) failure in the fusion between autophagosomes and lysosomes; or (v) defective lysosomal degradation. Thus, it is imperative to study the mechanism of autophagy in a variety of *in vivo* and *in vitro* model systems to gain a complete understanding of the role of autophagy in ALS. This knowledge would allow for tailoring of therapeutics to specifically target affected pathways.

### Superoxide Dismutase 1 (SOD1)

SOD1 is a highly conserved cytoplasmic and mitochondrial antioxidizing enzyme that is involved in scavenging and converting toxic superoxide radicals into reactive oxygen species (ROS) and hydrogen peroxide (H_2_O_2_; Wang et al., 2006). In 1993, the first genetic mutations were identified in the *SOD1* gene in individuals with fALS (Rosen et al., 1993) Further studies of fALS genomes has led to the discovery of more than 100 mutations in the *SOD1* gene. Currently, *SOD1* mutations account for approximately 12% of fALS cases and about 1% of sALS cases (Renton et al., 2014).

Despite being the first genetic link to ALS, the role of SOD1 in the pathogenesis of ALS is still an active area of investigation. Early studies suggested autophagy activation had a positive effect on cell proliferation and survival in mutant SOD1-mediated toxicity (Kabuta et al., 2006). A plausible mechanism came to light when it was observed that levels of the transcription factor, Transcription factor EB (TFEB), and one its key target genes, *BECN1*, were downregulated in spinal cord motor neurons of *SOD1*-ALS patients (Chen et al., 2015). *BECN1* encodes for Beclin-1, which is a key regulator of autophagy initiation; thus down-regulation of *BECN1* suggests reduced autophagy in these neurons. In support of this, overexpression of TFEB in cells expressing SOD1^G93A^ promoted autophagy (Chen et al., 2015). Overexpression of TFEB also increased cell proliferation, pointing to a direct correlation between autophagy induction and cell survival (Chen et al., 2015). Apart from TFEB, parkin has been implicated in K63-linked poly-ubiquitination of SOD1 mutant proteins (Yung et al., 2016). Interestingly, overexpression of parkin in cells expressing SOD1^A4V^ and SOD1^G93A^ appeared to ameliorate mutant protein toxicity through ubiquitination while having no effect on cells expressing wild-type SOD1. Furthermore, inhibition of the autophagy-lysosome pathway, but not UPS, inhibited the cytoprotective effects of parkin in SOD1 mutant cells, suggesting that increasing parkin levels aids in clearance of misfolded SOD1 aggregates through an upregulation of autophagy (Yung et al., 2016). It has been hypothesized that parkin facilitates misfolded SOD1 aggresome formation, a precursor to pathogenic insoluble aggregates, followed by K63-linked poly-ubiquitination. This attracts autophagy adaptor proteins, such as p62, resulting in degradation of the aggresomes through autophagy-lysosome machinery (Yung et al., 2016). In addition to failure in clearing SOD1 protein aggregates, autophagy also fails to clear damaged mitochondria that are a common pathological feature of SOD1-ALS. In spinal motor neurons derived from *SOD1*^G93A^ mice, there is a positive correlation between lysosomal deficits that alter endolysosomal trafficking, gross morphological defects in the mitochondria, and impaired degradation of the damaged mitochondria through mitophagy (Xie et al., 2015).

Early development of a SOD1 transgenic mouse model has made it possible to study autophagy function in SOD1-associated ALS, particularly in the context of disease progression. Mouse models reveal differential roles of autophagy. The high occurrence of autophagosomes, indicated by an increase in LC3-II levels, was the first evidence of increased autophagy in SOD1^G93A^ mutant animals (Xie et al., 2015). To further examine the importance of autophagy in SOD1-ALS, the disease phenotype was studied in the background of BECN1 deficiency (Tokuda et al., 2016). BECN1 is an important regulator of autophagy at the initiation step. *Becn1* haploinsufficient mice exhibited reduced autophagic activity (Nassif et al., 2014). Loss of BECN1 in mice expressing SOD1^G127X^ impaired autophagic flux and further exacerbated SOD1 aggregation. This was accompanied by an early onset and faster progression of the disease along with a significantly shorter lifespan in these animals (Tokuda et al., 2016). These phenotypes are also complemented by an increase in SOD1^G127X^ protein aggregates (Tokuda et al., 2016). In contrast, BECN1 deficiency in SOD1^G86R^ mice had the opposite effect on disease progression, with a significant delay in disease onset and increased animal lifespan (Nassif et al., 2014). Thus, despite the evidence in *in vitro* systems supporting reduced autophagy function in SOD1-ALS, *in vivo* systems indicate a more complex, distinct role of autophagy based on the particular SOD1 mutation. Interestingly, autophagy also seems to have differential roles at different stages of the disease. A group that investigated the effects of inducing autophagy by restricting food intake to emulate starvation conditions showed that activating autophagy at the onset of the disease, rather than late stage, had beneficial effects (Zhang et al., 2013).

Early symptomatic stages of the SOD1^G93A^ mouse model also exhibited impaired degradation of damaged mitochondria through mitophagy (Xie et al., 2015). The most prominent feature was observed in axon exit zones of the ventral root where there was accumulation of abnormal autolysosomes called multilamellar bodies (MLBs) and amphisome-like structures around damaged mitochondria with elevated ROS production. This model indicates autophagy dysfunction that is most likely caused by defects in endosomal trafficking (Xie et al., 2015). Improving the endosomal trafficking through overexpression of snapin, an adaptor protein that coordinates dynein-driven retrograde transport of late endosomes, rescues mitochondrial pathology and motor neuron loss in the mice (Xie et al., 2015).

### TAR DNA-Binding Protein 43 (TDP-43)

The transactive DNA-binding protein 43 (TDP-43) is a heterogeneous nuclear protein that is a prominent feature of ubiquitinated cytoplasmic inclusions observed in ALS and FTLD patients (Neumann et al., 2006). TDP-43 is primarily involved in RNA metabolism, including, mRNA and miRNA processing, alternative splicing, transport and stability (Scotter et al., 2015). Mutations in *TDP-43* contribute to 4%–5% of fALS and nearly 1% of sALS cases, and most of the mutations are concentrated in the C-terminus of the protein (Millecamps et al., 2010). TDP-43 immunopositive inclusions were found in the motor cortices and spinal cords of nearly 97% of fALS and sALS patients, suggesting an important consequence for the aggregation of the protein into inclusions (Sreedharan et al., 2008; Qin et al., 2014).

A plausible mechanism is that sequestration of TDP-43 in these aggregates prevents it from carrying out its function, which results in cellular toxicity. One of its target mRNAs codes for the autophagy-related protein, ATG7, thus TDP-43 may play a role in regulating autophagy (Bose et al., 2011). ALS-causing mutations in TDP-43 impair its ability to bind and stabilize ATG7 mRNA, thus leading to autophagy dysfunction (Bose et al., 2011). This indicates that TDP-43 aggregation in the cytoplasm causes autophagy impairment, or vice versa. Further evidence linking TDP-43 to autophagy regulation has pointed to the involvement of the transcription factor TFEB (Xia et al., 2016). In neuronal cells, one study reported that TDP-43 regulated autophagy by affecting the localization of TFEB, thus affecting the expression of its transcription targets that code for autophagy and lysosomal proteins (Xia et al., 2016). In addition, TDP-43 has been shown to help autophagosome-autolysosome fusion through regulation of DCTN1 levels (Xia et al., 2016). This further supports the model that TDP-43 regulates autophagy, and thus regulates its own turnover. Aggregates containing caspase-cleaved forms of the protein, TDP-25 and TDP-35, are a common feature in the brains of some ALS patients (Neumann et al., 2006). Importantly, these small fragments of TDP-43 have been shown to cause toxicity when expressed in cellular and animal models, such as *Drosophila* and mouse (Lagier-Tourenne et al., 2010; Casci and Pandey, 2015). When autophagy was chemically inhibited using 3MA (3-Methyladenine), the levels of TDP-25 and TDP-35 increased due to decreased turnover of these fragments, further supporting the notion that TDP-43 protein fragments are managed by autophagy pathway (Brady et al., 2011). In contrast, administration of rapamycin or trehalose, autophagy inducers, increased fragment turnover and reduced cytoplasmic mislocalization and aggregation of TDP-43 (Caccamo et al., 2009; Wang et al., 2010). While the specific mechanism of TDP-43-induced autophagy dysfunction is still under study, an overall upregulation of autophagy seems to be an area of therapeutic potential.

The effect of inducing autophagy at different stages of the ALS pathogenesis has been explored in a TDP-43 proteinopathy mouse model. In an FTLD-U mouse model that forms TDP-43 aggregates, rapamycin administration at early stages rescued the motor deficits and neuronal loss, while at a later stage it effectively reduced TDP-43 ubiquitinated inclusions (Wang I.-F. et al., 2013). Autophagy dysregulation has also been reported in a study examining the effects of TDP-25 overexpression and aggregation *in vivo* (Caccamo et al., 2015). Similar to *in vitro* studies, the TDP-25 mouse model showed impaired protein turnover of TDP-25 within aggregates. These mice exhibited reduced levels of autophagy-initiation markers, indicating that impaired protein turnover was most likely a consequence of reduced autophagy (Caccamo et al., 2015). However, the effect of inducing autophagy in this mouse model has not yet been investigated. Similar to mice, overexpression of endogenous TDP-43 in *Drosophila* resulted in formation of aggregates within neurons, accompanied by diminished lifespan and locomotive defects (Cheng C.-W. et al., 2015). These phenotypes were rescued by inducing autophagy, further supporting the protective effects of autophagy induction *in vivo* (Cheng C.-W. et al., 2015).

### Fused in Sarcoma (FUS)

FUS belongs to the FET family of proteins that, like TDP-43, is also a heterogeneous ribonuclear protein with roles in mRNA transcription, transport, trafficking, and alternative splicing (Deng et al., 2014). Mutations in *FUS* were discovered in fALS cases in 2009. Since then, over 40 mutations in *FUS* have been reported (Kwiatkowski et al., 2009; Vance et al., 2009). Collectively, these mutations account for about 4% of fALS and 1% of sALS cases (Deng et al., 2014). A unique feature of *FUS* mutations is the significant effect of heterogeneity in prognosis of the disease. For example, the P525L mutation causes juvenile (early twenties) ALS, whereas mutations such as R521C and R518K are associated with late-onset disease presentation (forties to sixties; Deng et al., 2014).

In addition to ALS-associated cytoplasmic mislocalization and formation of aggregates, FUS is also known to be a major component of cytoplasmic stress granules. In the case of *FUS* mutations, cellular stress can initiate the formation of mRNA and protein-containing dynamic foci called stress granules (Bosco et al., 2010). FUS proteins that mislocalize to the cytoplasm are trapped into these stress granules and are not released even after the stressor is removed (Daigle et al., 2016). In addition, these FUS-containing stress granules co-localize to autophagosomes (Ryu et al., 2014). In primary neurons expressing a mutant form of FUS, a reduction in autophagy increased the number of FUS-positive stress granules. This effect was reversed in these neurons when autophagy was induced using rapamycin (Ryu et al., 2014). This suggests an important role for autophagy in the formation of pathogenic stress granules and recruitment of FUS protein into these granules. One study provided evidence of *FUS*^P525L^ and *FUS*^R522G^ mutations directly impairing autophagy in neuronal cell lines and primary cortical neurons (Soo et al., 2015). Mutant FUS expression resulted in formation of fewer omegasomes, a precursor to autophagososomes, compared to expression of wild-type FUS. In addition, the early autophagosomes formed in mutant FUS-expressing cells recruited lesser ATG9 and lipidated LC3-II, both of which are required for autophagy initiation and elongation (Soo et al., 2015). This suggests that FUS mutations have a negative impact in the early stages of autophagosome synthesis, thus inhibiting autophagy. Interestingly, overexpression of Rab1 in these cells rescued the stress granule, omegasome, and autophagosome pathologies. Rab1 is normally involved in the trafficking of autophagosomes to lysosomes during autophagy. Restoration of autophagy upon Rab1 overexpression in mutant FUS-expressing cells suggests that mutant FUS might also be responsible for disrupting autophagosome-lysosome fusion through perturbation of Rab1 function (Soo et al., 2015). Further investigation into the role of autophagy in *FUS*-ALS could benefit from development of *in vivo* models that show incorporation of FUS into cytoplasmic stress granules and the subsequent effect of upregulation of autophagy.

### Chromosome 9 Open Reading Frame 72 (C9orf72)

Expansion of GGGGCC intronic HREs in the *C9orf72* gene is the most commonly identified cause of ALS, occurring in approximately 40% of fALS and 7% of sALS cases (Majounie et al., 2012). The pathogenic mechanisms that cause *C9orf72* HRE-mediated toxicity in motor neurons of ALS patients are still heavily under investigation. Three potential mechanisms have so far come to light: haploinsufficiency caused by loss-of-function of the *C9orf72* gene, sense and antisense RNA foci toxicity, and accumulation of toxic DPR proteins, which are closely associated with TDP-43 aggregation (Mackenzie et al., 2013; Mori et al., 2013).

Although multiple studies have investigated the pathogenesis of C9orf72-associated ALS, little is known about the function of the endogenous protein. In primary mouse cortical neurons, depletion of C9orf72 lead to p62 aggregate formation, which is one of primary pathological features of ALS/FTLD patients with expanded *C9orf72* repeats. These data suggest that C9orf72 may have a direct role in maintaining protein homeostasis (Sellier et al., 2016; Sullivan et al., 2016). In human spinal cord motor neurons of patients with *C9orf72* expansion, the protein co-localized with Rab7 and Rab11 at a higher frequency than controls (Farg et al., 2014). Rab7 and Rab11 are members of small GTPase family of proteins that facilitate autophagy by assisting in endo-lysosomal trafficking (Wang et al., 2011; Szatmári et al., 2014). Consistent with this, there has been a recent bout of evidence implicating C9orf72 as a GEF (guanine nucleotide exchange factor) effector for Rab GTPases (Sellier et al., 2016; Sullivan et al., 2016; Webster et al., 2016). This implicates a direct role for endogenous C9orf72 in autophagy through its GEF effector activity. Another study suggested that C9orf72 forms a complex with two other proteins, Smith-Magenis Syndrome Chromosome Region, Candidate 8 (SMCR8) and WD repeat domain 41 (WDR41), and this complex acted as a GEF for Rab GTPases such as Rab39b, Rab8a and Rab1a, thereby facilitating their role in endocytic trafficking in autophagy (Sullivan et al., 2016). In an alternative mechanism, C9orf72 bound to Rab1a and ULK1 (of the autophagy initiation complex) which aided Rab1a-dependent trafficking of the autophagy initiation complex to the phagophore and subsequent formation of autophagosomes (Webster et al., 2016). Loss of C9orf72 also failed to initiate autophagy in neurons treated with autophagy inducer (Webster et al., 2016). These data suggest that C9orf72 plays a critical role in the autophagy pathway, more specifically, in the initiation stage of autophagy. Together this presents a loss-of-function model for *C9orf72* repeat expansion–associated ALS/FTLD in which the *C9orf72* HRE perturbs its function in the Rab cascade during autophagy initiation. This, in turn, could affect the downstream processes of autophagy and clearance of toxic aggregates, ultimately leading to neurodegeneration.

On the other hand, contrary evidence points to increased autophagic flux and lysosomal degradation in both *in vitro* and *in vivo* models of C9orf72 deficiency (Ugolino et al., 2016). This particular study observed that loss of C9orf72 lead to reduced mTOR signaling and increased TFEB activity, both of which could contribute to autophagy activation. Nutrient starvation of *C9orf72*-knockout mice resulted in increased autophagic flux accompanied by increased levels of TFEB and its lysosomal targets (Ugolino et al., 2016). To account for the apparent contradictory roles of C9orf72 haploinsufficiency in autophagy, the authors argued that C9orf72 may have a multifunctional role where it regulates autophagy initiation and also autophagic flux (Ugolino et al., 2016). All studies so far have utilized cellular and animal models in which *C9orf72* has been knocked down or knocked out. Further studies in either patient-derived iPSC motor neurons (Almeida et al., 2013) that exhibit *C9orf72*-haploinsufficiency or other models that express the *C9orf72* ALS-associated GGGGCC expansion (Haeusler et al., 2016) could give more insight into the role of autophagy in *C9orf72-ALS*.

### Ubiquilin 2 (UBQLN2)

UBQLN2 is a multi-domain member of the UBL-UBA family of proteins, in which the UBL (ubiquitin-like) and UBA (ubiquitin-associated) domains facilitate their dual role in autophagy and ubiquitin-proteasome systems (Osaka et al., 2016). The UBA domain facilitates binding of UBQLN2 to polyubiquitinated proteins, and the UBL domain facilitates delivery of ubiquitinated substrates to proteasomes (Osaka et al., 2016). In autophagy, ubiquilins have been shown to act as autophagy receptors by recruiting autophagosomes to polyubiquitinated aggregates through interaction with LC3, thus aiding in aggregate clearance (Rothenberg et al., 2010). Mutations in *UBQLN2* were discovered in patients with fALS, providing a direct association between disease pathogenesis and impaired clearance of toxic aggregates (Osaka et al., 2016).

In cell culture systems, evidences for the role of UBQLN2 and the consequences of ALS-associated mutations in autophagy have been limited. UBQLN2 has been implicated in affecting protein turnover, including TDP-43, since overexpression of ALS-associated *UBQLN2* mutations in neuronal cells increased TDP-43 levels and promoted aggregation (Osaka et al., 2016). The same study also showed that the *UBQLN2*^1Vm^ mutation disrupted its interaction with autophagy proteins ATG9 and ATG16L1 (Osaka et al., 2016). Both proteins are present on autophagosome surfaces and facilitate binding of autophagosomes to lysosome. This indicates that *UBQLN2* mutations possibly disrupt its incorporation into autophagosomes.

Transgenic rats expressing *UBQLN2*^P497H^ recapitulated features of *UBQLN2*-related ALS, including protein aggregates that contained the mutant UBQLN2, motor neuron loss, cortex structure deformations, and progressive accumulation of p62 and LC3-II proteins within the aggregates (Wu et al., 2015; Huang et al., 2016). Aggregates formed by the mutant protein within rat neurons also sequestered endogenous UBQLN2, suggesting it may contribute to toxicity via a dominant-negative mechanism (Wu et al., 2015). The rats also had a lower number of early endosomes, which indicated defects in the early endosome pathway. While these results indicate defective protein degradation, the specific role of UBQLN2 mutations in autophagy remains unclear. One hypothesis is that UBQLN2 interacts with OPTN at endosomal vesicles and act in concert to facilitate endosome trafficking during autophagy (Osaka et al., 2015). However, the interaction has not been investigated in the context of *UBQLN2* ALS-associated mutations. Considering its dual role in UPS and autophagy, it is yet to be determined if the ALS-associated mutations in *UBQLN2* or *OPTN* affect protein degradation predominantly through one, or both, pathways.

### Optineurin (OPTN)

OPTN is a cytoplasmic protein that plays an important role in vesicle trafficking, autophagy and NF-kB signal transduction (Ying and Yue, 2012). *OPTN* mutations constitute about 1–4% of fALS cases, with one of the mutations falling in the ubiquitin-binding UBAN domain of the protein (Li et al., 2016).

OPTN acts as an autophagy receptor whose role in degradation of damaged mitochondria has come to prominence. An important role for OPTN has been demonstrated in mitophagy, which is the selective degradation of damaged mitochondria by autophagy (Wong and Holzbaur, 2015). Under conditions of mitochondrial stress/damage, PINK1 recruits the E3-ubiquitin ligase, PARK1, to the outer mitochondrial membrane to ubiquitinate proteins. OPTN, an autophagy adapter, binds to the ubiquitinated protein and promotes autophagosome formation around the mitochondria, thus aiding in mitophagy (Wong and Holzbaur, 2015). This function of OPTN is inhibited by the ALS-associated mutation, E478G, which has deficient ubiquitin-binding abilities, and also by F178A, which has impaired LC3 recruitment (Wong and Holzbaur, 2015). This points toward a loss-of-function in autophagy for OPTN in ALS. This is further supported by the observation that damaged mitochondria were not degraded efficiently in OPTN-depleted cells (Wong and Holzbaur, 2015). A closer look at the kinetics of mitophagy in parkin-expressing cells revealed that after mitochondrial depolarization or ROS production, OPTN-mediated autophagosome formation around the mitochondria was preceded by TANK-binding Kinase 1 (TBK1) phosphorylation of OPTN (Moore and Holzbaur, 2016). This interaction was lost when *TBK1* carried the ALS-associated mutation, E696K. A similar effect was observed for OPTN mutants, E478G and R398X, resulting in failed mitophagy (Wong and Holzbaur, 2015; Moore and Holzbaur, 2016). However, there is conflicting data that points to no evident mitophagy defects 24 h post-mitochondrial depolarization even in the case of OPTN deficiency (Lazarou et al., 2015). This suggests that there might be compensatory mechanisms at play, which contrasts with the theory of loss-of-function in OPTN-ALS.

In addition to playing a role in mitophagy, OPTN might also regulate autophagic flux (Shen et al., 2015). A study investigating the link between OPTN and UBQLN2 autophagy receptors found that both proteins co-localized to the same cellular component on recycling endosomes. Recycling endosomes are thought to contribute to autophagosome formation (Longatti and Tooze, 2012). ALS-linked mutations in both proteins affected the formation of recycling endosomes, suggesting that these mutations affected autophagosome formation. Conversely, another study investigating the effects of *OPTN* mutations within the ubiquitin-binding domain reported that *OPTN* mutations affected autophagosome turnover by inhibiting fusion with lysosomes rather than affecting autophagosome formation itself (Shen et al., 2015). A possible explanation could be that OPTN plays a dual role where it facilitates autophagosome fusion with lysosomes and also interacts with UBQLN2 on the surface of recycling endosomes to contribute to autophagosome formation.

### Sequestosome 1/p62 (SQSTM1/p62)

SQSTM1, or p62, is an autophagy receptor protein that also has other roles in NF-kB signaling, Keap1/Nrf2 activation, and apoptosis (Rea et al., 2013). SQSTM1/p62 is also known to interact with mTOR and regulates the translocation of mTOR to lysosomes (Duran et al., 2011). SQSTM1 has been associated with many other neurodegenerative diseases, including Alzheimer’s, Parkinson’s and Huntington disease, where p62/SQSTM1 is a predominant component of the toxic inclusions formed in the neurons. This indicates that SQSTM1 plays an important role in clearance of the inclusions (Wooten et al., 2006).

Mutations in *SQSTM1* have been discovered in rare cases of individuals with ALS/FTLD, and a majority of the mutations are associated with defective ubiquitin-recognition by the UBA domain of the protein (Rea et al., 2013). Another domain of interest with ALS/FTLD-linked mutations is the LC3-interacting region (LIR) domain, which is responsible for recruitment of autophagosomes to ubiquitinated substrates and subsequent degradation by autophagy (Rea et al., 2013). Absence of SQSTM1/p62 has been linked to defects in mitophagy owing to compromised autophagosome formation following mitochondrial depolarization (Haack et al., 2016). The effect SQSTM1/p62 loss-of-function has also been tested *in vivo* in zebrafish where knockdown of *sqstm1* led to development of abnormal motor behavior and disrupted arborization and shortening of motor neurons (Lattante et al., 2015). The neurodegenerative phenotypes were rescued with rapamycin administration, suggesting that loss of SQSTM1/p62 has deleterious effects through disruption of autophagy. In cell culture, the ALS-associated mutation in the LC3-binding domain of the protein, L341V, resulted in failed LC3 binding and incorporation of autophagic cargo into phagophores (Goode et al., 2016). This phenotype was also rescued by rapamycin, again supporting a pathogenic role for autophagy in *SQSTM1*-ALS (Goode et al., 2016). *In vitro* and *in vivo* studies thus far have leaned more towards a loss-of-function hypothesis for *SQSTM1* mutations, where the protein’s role in degradation pathways could be disrupted by the mutations. However, analysis of protein expression in spinal cord of patients with mutations in *SQSTM1* reveals an increase in the level of proteins, suggesting a gain-of-function (Teyssou et al., 2013). It remains to be determined if *SQSTM1* mutations in ALS/FTLD acts via a loss- or gain-of-function mechanism, as well as its consequence on autophagy regulation.

### Dynactin (DCTN1)

DCTN1 is an important protein in the retrograde transport of vesicles and organelles along microtubules via interaction with the motor protein, dynein (Laird et al., 2008). *DCTN1* mutations are found only in a very small subset of patients with ALS (Münch et al., 2004). Nevertheless, studies so far implicate a strong role for *DCTN1* mutations in vesicle transport defects in autophagy.

The ALS-associated mutation, G59S, impairs autophagic clearance of aggregated proteins and alters vesicular trafficking that manifests as accumulation of autophagosomes in motor neurons in mice (Laird et al., 2008). The dynamics of disrupted endocytic trafficking were better observed in a *dctn1*-deficient *C. elegans* model. The *dctn1*-deficient worms exhibited motor neuronal defects, decreased lifespan, decreased speed in movement, and axonal degeneration (Ikenaka et al., 2013). Live cell imaging of these mutant animals revealed impaired autophagosome transport and accumulation of untransported autophagosomes in the areas of axonal defects (Ikenaka et al., 2013). Moreover, induction of autophagy in this model through starvation, as well as rapamycin administration, strongly ameliorated the motor dysfunction and increased the frequency of autophagosome movement (Ikenaka et al., 2013). While these phenotypes could be interpreted as dysregulation in autophagosome transport, it is important to note that DCTN1 is an essential protein involved in transport of various cargoes and thus the effects observed could just be a by-product of defective cargo-transport machinery.

## Autophagy: Converging Point for Other Pathogenic Mechanisms

When examining age-related neurodegeneration in ALS, it is important to consider the crosstalk between autophagy and cellular dysfunctions in ALS that may contribute to autophagy dysregulation. Growing evidence supports the notion of potential crosstalk between different pathways in ALS and it is very likely that other biological pathways may directly or indirectly impact autophagy, and vice versa. The mechanisms that interact with autophagy in neurons could contribute to autophagy dysregulation in ALS in multiple ways—(i) either the pathways activate autophagy downstream and this crosstalk is perturbed in ALS; (ii) the downstream activation of autophagy in these pathways could overwhelm the autophagy pathway, resulting in toxic effect; or (iii) the other pathogenic mechanisms work in conjunction with a failed autophagy system, pushing the cells over the edge and thus causing neurodegeneration. Nevertheless, investigation of the crosstalk between the mechanisms may elucidate the role of autophagy itself by drawing attention to molecular links that could be further studied for determining early disease therapeutic interventions.

### Endoplasmic Reticulum (ER) Stress

**Figure 1:**
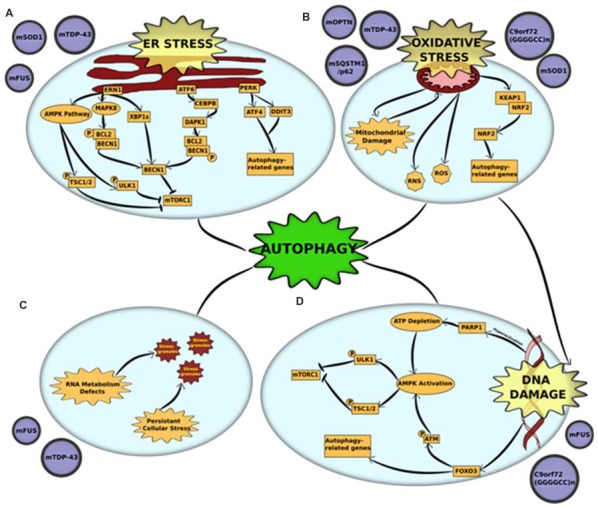
Cross-talk between autophagy and other pathogenic mechanisms in amyotrophic lateral sclerosis (ALS) **(A)** endoplasmic reticulum (ER) stress and unfolded protein response (UPR) is triggered by mutations in superoxide dismutase 1 (mSOD1), TAR DNA-binding protein 43 (TDP-43) and fused in sarcoma (FUS). Accumulation of misfolded mutant proteins activates either one or all three UPR pathways—protein kinase RNA-like endoplasmic reticulum kinase (PERK), endoplasmic reticulum to nucleus signaling 1 (ERN1) and activating transcription factor 6 (ATF6). Each of the pathways stimulate autophagy through downstream activators. ERN1 activates autophagy through three pathways—(i) the AMPK pathway phosphorylates and activates two proteins—ULK1 and tuberous sclerosis protein 1/2 (TSC1/2)—both of which inhibit mammalian target of rapamycin complex 1 (mTORC1) which leads to initiation of autophagy; (ii) ERN1 activates mitogen-activated protein kinase 8 (MAPK8), which uses its kinase activity to phosphorylate B-cell lymphoma 2 (BCL2) which is in a complex with Beclin-1 (BECN1). This phosphorylation disrupts the complex and releases BECN1, which in turn activates autophagy through mTORC1 inhibition; (iii) ERN1 also acts an endoribonuclease to splice XBP1. The spliced version of XBP1 (XBP1s) promotes expression of BECN1. The UPR activator ATF6 activates the transcription factor CCAAT/enhancer-binding protein beta (CEBPB) that promotes expression of the kinase death-associated protein kinase 1 (DAPK1). DAPK1 phosphorylates BECN1 and thus breaks it from its complex with BCL2. The free BECN1 now activates autophagy through mTORC1 inhibition. The third UPR activator, PERK, activates the expression of two stress response genes—activating transcription factor 4 (ATF4) and DNA damage inducible transcript 3 (DDIT3). These genes promote the transcription of autophagy-related genes (ATGs) that activate the process. **(B)** Oxidative stress and mitochondrial dysfunction are complimentary mechanisms that are triggered by mutations in optineurin (mOPTN), TDP-43, SQSTM1/p62, SOD1 and C9orf72 GGGGCC expansion. Mitochondrial dysfunction is the primary source of reactive oxygen and reactive nitrogen species (ROS and RNS) that are directly implicated in activating autophagy. An alternative mechanism is through the Kelch-like ECH-associated protein 1 (KEAP1)-nuclear factor erythroid 2-related factor 2 (NRF2) pathways. Oxidative stress KEAP1-NRF2 complex is disrupted. This leaves NRF2 free to translocate to the nucleus where it induces the expression of several autophagy-related genes. Autophagy also acts to clear damaged mitochondria through an organelle-specific mechanism called mitophagy. **(C)** RNA metabolism defect is a major pathological outcome of mutations in RNA-binding proteins, especially FUS and TDP-43. These proteins are major components of stress granules and mutations in low complexity domains of these proteins alter the phase of stress granules to give them a complex liquid state. This alters the dynamics of stress granules, in that, they are not cleared and potentially act as primers for protein aggregates. Autophagy is a crucial mechanism involved in the clearance of stress granules under normal conditions and dysfunction in autophagy could be linked to altered stress granule dynamics. **(D)** DNA damage, either caused as a direct result of pathogenic mutation or triggered by oxidative stress, is a common pathological feature of mutations in FUS and C9orf72 GGGGCC expansion. DNA damage activates autophagy through activation of AMPK pathway. The AMPK pathway phosphorylates and activates two proteins, ULK1 and TSC1/2, both of which independently act as mTORC1 inhibitors. DNA damage activates the AMPK pathways through: (i) Hyperactivation of PARP1, which leads to a depletion of NAD^+^ reserves in the cell. This, in turn, depletes ATP and thus activates AMPK; or (ii) FOXOa3 dissociation from DNA that allows it to interact with ATM. Activation of ATM by phosphorylation leads to downstream activation of AMPK pathway. Alternatively, FOXOa3 also acts as a transcription factor that regulates expression of autophagy-related genes.

The endoplasmic reticulum (ER) is a crucial organelle in which both membrane proteins and secreted proteins are synthesized and modified for protein folding. In a cell, more than one-third of the proteins synthesized are misfolded (Guerriero and Brodsky, 2012). Disruption in the protein-folding capacity of the ER and/or increased accumulation of misfolded proteins leads to ER stress. To restore protein homeostasis, a mechanism called the unfolded protein response (UPR) is triggered to decrease the load of unfolded proteins through various pathways (Guerriero and Brodsky, 2012). However, in certain cellular conditions, including but not restricted to nutrient deprivation and missense mutations, protein folding is enhanced, which results in severe ER stress (Guerriero and Brodsky, 2012). Prolonged ER stress causes an accumulation of unfolded or misfolded proteins, which eventually leads to apoptosis. There are several reports that support the presence of ER stress in the motor neurons of both fALS and sALS (Ilieva et al., 2007). *In silico* analyses of motor neurons from transgenic mutant SOD1 mice revealed ER stress to be an early pathological feature (Vlug et al., 2005; Atkin et al., 2006) In addition, ER stress has also been associated with *TDP-43*- and *FUS*-ALS (Vlug et al., 2005; Atkin et al., 2006). The mutant forms of these proteins induce either one or all three of the different UPR activators in the central nervous system—Protein kinase RNA-like endoplasmic reticulum kinase (PERK), Endoplasmic reticulum to nucleus signaling 1 (ERN1) and Activating transcription factor 6 (ATF6; Vlug et al., 2005; Atkin et al., 2006; Farg et al., 2012; Walker et al., 2013). Interestingly, each of the three UPR pathways has been shown to stimulate autophagy to relieve cellular stress or, in extreme cases, induce apoptosis (Figure 1A).

The PERK pathway works through downstream activation of stress response genes. Of these, Activating transcription factor 4 (ATF4) and DNA damage inducible transcript 3 (DDIT3), independently and together, promote transcription of several autophagy genes, including *MAP1LC3B, BECN1, ATG3, ATG12, ATG16L1, SQSTM1, Neighbor of Brca1 (NBR1), ATG7, ATG10, GABA Type A receptor-associated protein (GABARAP)* and *ATG5*, thereby inducing autophagy (B’chir et al., 2013). ATF4 expression was also upregulated in PERK-activated cells through phosphorylation of EIF2α, both of which were detected in their activated forms in mutant SOD1 models (Matus et al., 2013).

Following ER stress, ERN1 activates autophagy through more than one pathway. The first is through the AMP-activated protein kinase (AMPK) pathway, which induces autophagy through mammalian target of rapamycin (mTORC1) inhibition (Rashid et al., 2015). The second, more predominant pathway is via Mitogen-activated protein kinase 8 (MAPK8)-mediated phosphorylation of B-cell lymphoma 2 (BCL2). This disrupts BCL2’s interaction with BECN1, freeing it to activate autophagy (Rashid et al., 2015). ERN1 induces BECN1 activity also through an alternative mechanism that uses its endoribonuclease activity to produce spliced X-box-binding protein 1 (XBP1) that, in turn, promotes *BECN1* expression (Rashid et al., 2015).

ATF6 activator also induces autophagy through BECN1. The ATF6-associated transcription factor CCAAT/enhancer-binding protein beta (CEBPB) promotes expression of Death-associated protein kinase 1 (DAPK1; Kalvakolanu and Gade, 2012). Activated DAPK1 phosphorylates BECN1, stimulating dissociation of the phosphorylated BECN1 from its complex with BCL2. This frees BECN1 to induce autophagy (Kalvakolanu and Gade, 2012).

### Mitochondrial Dysfunction

Mitochondrial dysfunction in ALS can be manifested through multiple pathogenic mechanisms, including impaired oxidative phosphorylation complexes (OXPHOS), reduced respiration and ATP synthesis, disrupted calcium homeostasis and increased ROS production (Muyderman and Chen, 2014). The primary link between mitochondrial dysfunction and autophagy is through mitophagy (Zhang, 2013). The dysfunctional mitochondria are ubiquitinated by autophagy receptors, including OPTN, UBQLN2, VCP and TBK1 (Wong and Holzbaur, 2014, 2015; Guo et al., 2016; Hjerpe et al., 2016; Moore and Holzbaur, 2016). ALS-associated mutations in these proteins lead to disrupted clearance of damaged mitochondria. The role of impaired mitophagy in the pathogenesis of ALS has been discussed in the previous section. Mitochondrial dysfunction is also the primary source of ROS production, which leads to elevation of oxidative stress and subsequent oxidative stress-induced autophagy (Figure 1B).

### Oxidative Stress

Oxidative stress been firmly established in the etiology of ALS, caused by an imbalance between the production of ROS and other oxidants and elimination of these species by the cellular antioxidant defense system (D’Amico et al., 2013). Neurons use 10 times more oxygen than other tissues, making them especially vulnerable to oxidative stress (Lee et al., 2012). The main source of ROS in autophagy signaling occurs via mitochondrial damage/dysfunction, the role of which has been discussed elaborately in the previous section. The Keap1-Nrf2 pathway is another mechanism through which oxidative stress induces autophagy (Figure 1B). Under basal conditions, Keap1 negatively regulates Nrf2 through direct association. However, under oxidative stress, binding is disrupted and Nrf2 is free to translocate to the cytoplasm where it regulates the expression of multiple autophagy genes (Gan and Johnson, 2014). Keap1-Nrf2 pathway was altered in animal models of ALS as well as in post-mortem tissues derived from patients (Kanno et al., 2012). In addition, Keap1 immunoreactivity has been detected in the skein-like inclusions from the spinal cords of ALS patients, possibly through an interaction with p62/SQSTM1, a protein that has been observed in several ALS inclusions (Goode et al., 2016). Another mechanism of oxidative stress-induced autophagy in ALS is through stress granule formation and subsequent degradation of stress granules by autophagy (Finelli et al., 2015). In fact, when the oxidative stress resistance protein (Oxr1) was upregulated in neuronal cells, FUS and TDP-43’s cytoplasmic mislocalization and aggregation was significantly reduced (Finelli et al., 2015). This suggests an important pathogenic role of oxidative stress in stress granule formation in *FUS*- and *TDP-43*- associated ALS. In addition to acting through ROS, oxidative stress also has the potential to change the native conformations of proteins, potentially increasing their propensity to aggregate. Degradation of such protein aggregates through autophagy provides yet another link between oxidative stress and autophagy (Figure 1B).

### DNA Damage

Oxidative stress and DNA damage go hand in hand; oxidative stress results in years of accumulation of DNA damage. In ALS individuals—especially in the early stages of the disease—increased oxidative DNA damage has been observed, accompanied by a widespread increase in poly-adenosine diphosphate ribose polymerase (PARP1) and expression of enzymes involved in base-excision repair (Coppedè, 2011). When DNA is damaged, PARP1 binds to the DNA and polymerizes to form PAR (polyadenosine diphosphate ribose) chains. This signals the recruitment of various DNA damage repair proteins, including FUS (Coppedè, 2011). The role of FUS in DNA damage response/repair and the consequence of ALS-associated mutations on its function have been explored in a number of studies (Sama et al., 2014). ALS-associated *FUS*^R521C^ transgenic mice showed a robust increase in DNA damage repair defects in cortical and spinal motor neurons (Qiu et al., 2014). It has been suggested that DNA damage caused by double-stranded breaks stimulates wild-type FUS recruitment to DNA damage foci where it interacts with chromatin remodeling factor, HDAC1 (histone deacetylase 1). However, this interaction was shown to be impaired in FUS^R521C^, thus limiting its function in DNA damage repair (Wang W.-Y. et al., 2013; Qiu et al., 2014). Increased DNA damage pathology in ALS has also been successfully reproduced in human iPSC-derived motor neurons expressing mutant FUS (Higelin et al., 2016). In addition to FUS, a recent study observed an age-dependent increase in DNA damage pathology in motor neurons differentiated from iPSCs derived from C9orf72 patients. This was possibly through a mechanism involving mitochondrial dysfunction followed by increased oxidative stress, which led to an accumulation of DNA damage (Lopez-Gonzalez et al., 2016).

As the role of DNA damage in ALS and the potential to target DNA damage in early stages of the disease becomes increasingly prominent, it is interesting to note that DNA damage-repair acts as a cytoprotective mechanism by inducing DNA damage repair mechanisms, and also by inducing autophagy (Figure 1D). DNA damage leads to an induction of autophagy primarily through activation of the AMPK pathway (Eliopoulos et al., 2016). The AMPK pathway is responsible for downstream phosphorylation of two proteins—ULK1 and Tuberous sclerosis protein 1/2 (TSC1/2)—both of which are inhibitors of mTORC1 and subsequent autophagy initiation (Czarny et al., 2015). When DNA is damaged, hyperactivation of PARP1 leads to depletion of NAD^+^ reserves in the cell. This causes a decrease in ATP levels leading to downstream activation of AMPK pathway. An alternative pathway of AMPK activation occurs via dissociation of FOXO3 from DNA during DNA damage (Eliopoulos et al., 2016). Free FOXO3 interacts with ATM, which activates the AMPK pathway and leads to subsequent induction of autophagy (Figure 1D). FOXO3 also acts as a transcription factor that aids in autophagy activation by regulating transcription of ATGs, including *LC3* and *BNIP3* (Czarny et al., 2015).

### RNA Metabolism Defects

Cytoplasmic mislocalization and formation of aggregates in either the cytoplasm and/or the nucleus by RNA-binding proteins is a hallmark of multiple neurodegenerative disorders (Ugras and Shorter, 2012). ALS-associated RNA-binding proteins, including FUS, TDP-43, hnRNPA1, hnRNPA2B1 and MATR3, are all involved in RNA metabolism with roles in pre-mRNA splicing (Ugras and Shorter, 2012). These RNA-binding proteins translocate to the nucleus along with mRNA. Until mRNA translation, they sequester mRNA into membrane-less organelles called ribonucleoprotein granules (RNPs). There are two main types of RNPs: P-bodies and stress granules (Fan and Leung, 2016). Stress granules are formed in the cell under conditions of high stress and act to repress translation initiation of the mRNAs. These stress granules have a complex liquid state that arise via phase separation that is facilitated by the low complexity domains present in the RNA-binding proteins (Patel et al., 2015; Conicella et al., 2016; Boeynaems et al., 2017). A lot of ALS-related mutations occur in the low complexity domains. This could attribute to alterations in stress granule dynamics and result in irreversible stress granule formations that are primers for aggregation (Figure 1C). Disrupted assembly, disassembly, and clearance of stress granules has been associated with multiple models of ALS (Conicella et al., 2016; Boeynaems et al., 2017; Patel et al., 2015). Autophagy has been shown to be a crucial mechanism in the clearance of stress granules (Buchan et al., 2013). Inhibition of either autophagy or lysosome formation, or compromising VCP function through ALS-related mutations have all been shown to alter stress granule assembly and disassembly (Buchan et al., 2013; Seguin et al., 2014). In addition, stress granules that form under conditions of autophagy inhibition accumulate cellular components, such as defective ribosomal products (DRiPs) and 60s ribosomal subunits, that are not otherwise accumulated (Seguin et al., 2014). These, in turn, contribute to defective stress granule dynamics that persist in diseased cells, thus acting as seeds for aggregation.

## Therapeutic Implications

Autophagy-modulating drugs have been studied for treating various human neurodegenerative diseases as well as aging (Ballou and Lin, 2008; Díaz-Troya et al., 2008). The therapeutic potential of autophagy induction has been explored in different models of ALS. Rapamycin, shown to have a neuroprotective role in neurodegeneration, reduced aberrant protein aggregation in TDP-43 and SOD1 models of ALS (Wang I.-F. et al., 2013; Zhang et al., 2013). On the other hand, contradictory evidence in mice has shown that rapamycin worsens motor neuron degeneration and life span of SOD1 mutant mice (Zhang et al., 2011). This paradox suggests that rapamycin might have off-target effects that manifest in certain neurodegeneration models, highlighting the need for developing autophagy modulators with higher specificity. Use of mTOR-independent autophagy inducer, trehalose, has been shown to ameliorate SOD1 mutant toxicity and protein aggregation—albeit only in early stages of the disease—suggesting it may be a good therapeutic agent when administered in combination with other compounds (Zhang et al., 2014; Li et al., 2015). Another compound that has presented conflicting results is lithium carbonate, a drug that has been conventionally used to treat bipolar disorder (Machado-Vieira et al., 2009). One study investigating the *SOD1*^G93A^ mouse model of ALS found that lithium administration reduced motor neuron loss and increased survival (Pizzasegola et al., 2009). Other groups using similar experimental conditions have provided contrary evidence in which the mutant mice developed earlier disease onset and exhibited reduced lifespans (Fornai et al., 2008; Gill et al., 2009). While autophagy induction is a promising therapeutic strategy, these studies further support the need for developing more specific and targeted agents. In particular, high-throughput screens conducted in cells identified several novel small molecules that significantly modulate autophagy without leading to cytotoxicity (Zhang et al., 2007; Kuo et al., 2015).

The benefits of using high-specificity small molecule regulators of autophagy have been explored in several neurodegenerative diseases (Sarkar and Rubinsztein, 2008). In ALS, small molecule activators of autophagy have been shown to ameliorate toxicity in both TDP-43 and SOD1 mutant models of the disease. One notable study used a cell-based model to screen an *in silico* library that consisted of over a million potent autophagy activators (Barmada et al., 2014). The investigators identified two potential compounds that significantly stimulated autophagy: fluphenazine and methotrimeprazine. The compounds not only improved cell survival, but also reduced levels of TDP-43 and rescued its mislocalization (Barmada et al., 2014). Another study investigated the effects of the natural herb, berberine, which also exhibited therapeutic effects in TDP-43 proteinopathy through activation of autophagy (Chang et al., 2016). Berberine administration in a cell-based model increased metabolic rates and reduced aggregate formation of cleaved TDP-43 fragments (Chang et al., 2016). In addition to cell-based models, a different study used an FTLD-U mouse model of TDP-43 proteinopathy to study the effects of using small molecules to stimulate autophagy. This study used three chemical compounds—spermidine, tamoxifen and carbamazepine—that significantly improved the motor function of FTLD-U mice (Wang I.-F. et al., 2013). Activation of autophagy through the use of small molecules has also been shown to have therapeutic effects in SOD1 mutant mouse models. A natural compound called n-butylidenephthalide (n-BP) prolonged survival, improved motor functions, and reduced motor neuron loss in the spinal cords of *SOD1*-ALS mice (Hsueh et al., 2016). Interestingly, n-BP is known to regulate ER stress (Chiu et al., 2012), suggesting that n-BP may regulate autophagy in these mice through ER stress. Recently, the FDA approved a new drug called edaravone, also known as Radiciva, for the treatment of ALS (Yoshino and Kimura, 2006; Abe et al., 2014). The drug acts as a free radical scavenger and could thus significantly alleviate oxidative stress and delay neurodegeneration in ALS (Abe et al., 1988). This research opens up further avenues of modulating autophagy through targeting other pathogenic pathways that interact with autophagy, as described in the previous section.

## Future Directions and Conclusion

Dysregulation in autophagy is emerging as a major pathogenic hallmark of ALS. Most evidence point to a reduction in autophagy in diseased neurons and the subsequent therapeutic effects of inducing autophagy. One potential mechanism through which autophagy dysregulation could be caused is by a direct dysfunction of proteins involved in protein degradation. This hypothesis was solidified by the discovery of ALS-causing mutations in these proteins. An alternative mechanism could be through a combined effect of autophagy defects along with other cellular stresses that depend on autophagy for their alleviation. Prominently, phase separation defects caused by mutations in RNA-binding proteins with a low complexity domain allow them to aggregate into stress granules in diseased neurons. These stress granules act as a seed for self-aggregation of these proteins as well as interacting proteins. Over the life of a neuron, these cellular insults accumulate to the point of motor neuron death. Development of therapeutics that specifically target upstream cellular events could aid in alleviating cellular stress. A combination therapy with drugs that upregulate autophagy could be a promising therapeutic strategy for ALS.

## Author Contributions

NR wrote this review article. UBP edited and revised it.

## Conflict of Interest Statement

The authors declare that the research was conducted in the absence of any commercial or financial relationships that could be construed as a potential conflict of interest.

## Abbreviations

3MA: 3-methyladenenine
AMPK: AMP-activated protein kinase
ATF4: Activating transcription factor 4
ATF6: Activating transcription factor 6
ATG: Autophagy-related Gene
BCL2: B-cell lymphoma 2
BECN1: Beclin-1
CEBPB: CCAAT/enhancer-binding protein beta
DAPK1: Death-associated protein kinase 1
DDIT3: DNA damage inducible transcript 3
ERN1: Endoplasmic reticulum to nucleus signaling 1
FOXO3a: Forkhead box O3
GABARAP: GABA Type A receptor-associated protein
GEF: Guanine nucleotide exchange factor
KEAP1: Kelch-like ECH-associated protein 1
MAPK8: Mitogen-activated protein kinase 8
mC9orf72: Chromosome 9 open reading frame 9 GGGGCC expansion
mFUS: Mutations in fused in sarcoma
mOPTN: Mutations in optineurin
mSOD1: Mutations in superoxide dismutase 1
mSQSTM1: Mutations in sequestosome 1
mTDP-43: Mutations in TAR DNA-binding protein 43
mUBQLN2: Mutations in ubiquilin 2
mTORC1: Mammalian target of rapamycin complex 1
NBR1: Neighbor of Brca1
NF-κB: Nuclear factor kappa-light-chain-enhancer of activated B cells
NRF2: Nuclear factor erythroid 2-related factor 2
PARP1: Poly [ADP-ribose] polymerase 1
PERK: Protein kinase RNA-like endoplasmic reticulum kinase
RNS: Reactive nitrogen species
ROS: Reactive oxygen species
SMCR8: Smith-Magenis Syndrome Chromosome Region, Candidate 8
TBK1: TANK-binding Kinase 1
TFEB: Transcription factor EB
TSC1: Tuberous sclerosis protein 1/2
ULK1: Serine/threonine protein kinase ULK1
VPS34: Class III PI 3-kinase
WDR41: WD repeat domain 41
XBP1: X-box-binding protein 1.

## References

[B1] Abe K. Itoyama Y. Sobue G. Tsuji S. Aoki M. Doyu M.. (2014). Confirmatory double-blind, parallel-group, placebo-controlled study of efficacy and safety of edaravone (MCI-186) in amyotrophic lateral sclerosis patients. Amyotroph. Lateral Scler. Frontotemporal Degener. 15, 610–617. 10.3109/21678421.2014.959024 25286015 PMC4266079

[B2] Abe K. Yuki S. Kogure K. (1988). Strong attenuation of ischemic and postischemic brain edema in rats by a novel free radical scavenger. Stroke 19, 480–485. 10.1161/01.str.19.4.480 2834836

[B4] Almeida S. Gascon E. Tran H. Chou H. J. Gendron T. F. Degroot S.. (2013). Modeling key pathological features of frontotemporal dementia with *C9ORF72* repeat expansion in iPSC-derived human neurons. Acta Neuropathol. 126, 385–399. 10.1007/s00401-013-1149-y 23836290 PMC3753484

[B5] Al-Sarraj S. King A. Troakes C. Smith B. Maekawa S. Bodi I.. (2011). p62 positive, TDP-43 negative, neuronal cytoplasmic and intranuclear inclusions in the cerebellum and hippocampus define the pathology of *C9orf72*-linked FTLD and MND/ALS. Acta Neuropathol. 122, 691–702. 10.1007/s00401-011-0911-2 22101323

[B6] Arai T. Hasegawa M. Akiyama H. Ikeda K. Nonaka T. Mori H.. (2006). TDP-43 is a component of ubiquitin-positive tau-negative inclusions in frontotemporal lobar degeneration and amyotrophic lateral sclerosis. Biochem. Biophys. Res. Commun. 351, 602–611. 10.1016/j.bbrc.2006.10.093 17084815

[B7] Ash P. E. Bieniek K. F. Gendron T. F. Caulfield T. Lin W. L. Dejesus-Hernandez M.. (2013). Unconventional translation of *C9ORF72* GGGGCC expansion generates insoluble polypeptides specific to c9FTD/ALS. Neuron 77, 639–646. 10.1016/j.neuron.2013.02.004 23415312 PMC3593233

[B8] Atkin J. D. Farg M. A. Turner B. J. Tomas D. Lysaght J. A. Nunan J.. (2006). Induction of the unfolded protein response in familial amyotrophic lateral sclerosis and association of protein-disulfide isomerase with superoxide dismutase 1. J. Biol. Chem. 281, 30152–30165. 10.1074/jbc.M603393200 16847061

[B9] Balch W. E. Morimoto R. I. Dillin A. Kelly J. W. (2008). Adapting proteostasis for disease intervention. Science 319, 916–919. 10.1126/science.1141448 18276881

[B10] Ballou L. M. Lin R. Z. (2008). Rapamycin and mTOR kinase inhibitors. J. Chem. Biol. 1, 27–36. 10.1007/s12154-008-0003-5 19568796 PMC2698317

[B11] Barmada S. J. Serio A. Arjun A. Bilican B. Daub A. Ando D. M.. (2014). Autophagy induction enhances TDP43 turnover and survival in neuronal als models. Nat. Chem. Biol. 10, 677–685. 10.1038/nchembio.1563 24974230 PMC4106236

[B12] Basso M. Massignan T. Samengo G. Cheroni C. De Biasi S. Salmona M.. (2006). Insoluble mutant SOD1 is partly oligoubiquitinated in amyotrophic lateral sclerosis mice. J. Biol. Chem. 281, 33325–33335. 10.1074/jbc.m603489200 16943203

[B13] Bäumer D. Hilton D. Paine S. M. Turner M. R. Lowe J. Talbot K.. (2010). Juvenile ALS with basophilic inclusions is a FUS proteinopathy with FUS mutations. Neurology 75, 611–618. 10.1212/WNL.0b013e3181ed9cde 20668261 PMC2931770

[B14] B’chir W. Maurin A. C. Carraro V. Averous J. Jousse C. Muranishi Y.. (2013). The eIF2α/ATF4 pathway is essential for stress-induced autophagy gene expression. Nucleic Acids Res. 41, 7683–7699. 10.1093/nar/gkt563 23804767 PMC3763548

[B15] Boeynaems S. Bogaert E. Kovacs D. Konijnenberg A. Timmerman E. Volkov A.. (2017). Phase separation of *C9orf72* dipeptide repeats perturbs stress granule dynamics. Mol. Cell 65, 1044.e5–1055.e5. 10.1016/j.molcel.2017.02.013 28306503 PMC5364369

[B16] Boland B. Kumar A. Lee S. Platt F. M. Wegiel J. Yu W. H.. (2008). Autophagy induction and autophagosome clearance in neurons: relationship to autophagic pathology in Alzheimer’s disease. J. Neurosci. 28, 6926–6937. 10.1523/JNEUROSCI.0800-08.2008 18596167 PMC2676733

[B17] Bosco D. A. Lemay N. Ko H. K. Zhou H. Burke C. Kwiatkowski T. J. Jr.. (2010). Mutant FUS proteins that cause amyotrophic lateral sclerosis incorporate into stress granules. Hum. Mol. Genet. 19, 4160–4175. 10.1093/hmg/ddq335 20699327 PMC2981014

[B18] Bose J. K. Huang C. C. Shen C. K. (2011). Regulation of autophagy by neuropathological protein TDP-43. J. Biol. Chem. 286, 44441–44448. 10.1074/jbc.m111.237115 22052911 PMC3247982

[B19] Brady O. A. Meng P. Zheng Y. Mao Y. Hu F. (2011). Regulation of TDP-43 aggregation by phosphorylation and p62/SQSTM1. J. Neurochem. 116, 248–259. 10.1111/j.1471-4159.2010.07098.x 21062285

[B20] Bruijn L. I. Houseweart M. K. Kato S. Anderson K. L. Anderson S. D. Ohama E.. (1998). Aggregation and motor neuron toxicity of an ALS-linked SOD1 mutant independent from wild-type SOD1. Science 281, 1851–1854. 10.1126/science.281.5384.1851 9743498

[B21] Buchan J. R. Kolaitis R. M. Taylor J. P. Parker R. (2013). Eukaryotic stress granules are cleared by autophagy and Cdc48/VCP function. Cell 153, 1461–1474. 10.1016/j.cell.2013.05.037 23791177 PMC3760148

[B22] Caccamo A. Majumder S. Deng J. J. Bai Y. Thornton F. B. Oddo S. (2009). Rapamycin rescues TDP-43 mislocalization and the associated low molecular mass neurofilament instability. J. Biol. Chem. 284, 27416–27424. 10.1074/jbc.M109.031278 19651785 PMC2785671

[B23] Caccamo A. Shaw D. M. Guarino F. Messina A. Walker A. W. Oddo S. (2015). Reduced protein turnover mediates functional deficits in transgenic mice expressing the 25 kDa C-terminal fragment of TDP-43. Hum. Mol. Genet. 24, 4625–4635. 10.1093/hmg/ddv193 26002100

[B24] Carra S. Crippa V. Rusmini P. Boncoraglio A. Minoia M. Giorgetti E.. (2012). Alteration of protein folding and degradation in motor neuron diseases: implications and protective functions of small heat shock proteins. Prog. Neurobiol. 97, 83–100. 10.1016/j.pneurobio.2011.09.009 21971574

[B25] Casci I. Pandey U. B. (2015). A fruitful endeavor: modeling ALS in the fruit fly. Brain Res. 1607, 47–74. 10.1016/j.brainres.2014.09.064 25289585 PMC4385417

[B26] Chang C.-F. Lee Y.-C. Lee K.-H. Lin H.-C. Chen C.-L. Shen C. J.. (2016). Therapeutic effect of berberine on TDP-43-related pathogenesis in FTLD and ALS. J. Biomed. Sci. 23:72. 10.1186/s12929-016-0290-z 27769241 PMC5073438

[B27] Chen Y. Liu H. Guan Y. Wang Q. Zhou F. Jie L.. (2015). The altered autophagy mediated by TFEB in animal and cell models of amyotrophic lateral sclerosis. Am. J. Transl. Res. 7, 1574–1587. 26550457 PMC4626419

[B28] Cheng C.-W. Lin M. J. Shen C. K. (2015). Rapamycin alleviates pathogenesis of a new drosophila model of ALS-TDP. J. Neurogenet. 29, 59–68. 10.3109/01677063.2015.1077832 26219309

[B29] Cheng X.-T. Zhou B. Lin M. Y. Cai Q. Sheng Z. H. (2015). Axonal autophagosomes recruit dynein for retrograde transport through fusion with late endosomes. J. Cell Biol. 209, 377–386. 10.1083/jcb.201412046 25940348 PMC4427784

[B30] Chiu S. C. Chen S. P. Huang S. Y. Wang M. J. Lin S. Z. Harn H. J.. (2012). Induction of apoptosis coupled to endoplasmic reticulum stress in human prostate cancer cells by N-butylidenephthalide. PLoS One 7:e33742. 10.1371/journal.pone.0033742 22470469 PMC3314677

[B31] Conicella A. E. Zerze G. H. Mittal J. Fawzi N. L. (2016). ALS mutations disrupt phase separation mediated by α-helical structure in the TDP-43 low-complexity C-terminal domain. Structure 24, 1537–1549. 10.1016/j.str.2016.07.007 27545621 PMC5014597

[B32] Coppedè F. (2011). An overview of DNA repair in amyotrophic lateral sclerosis. ScientificWorldJournal 11, 1679–1691. 10.1100/2011/853474 22125427 PMC3201689

[B33] Crippa V. Carra S. Rusmini P. Sau D. Bolzoni E. Bendotti C.. (2010). A role of small heat shock protein B8 (HspB8) in the autophagic removal of misfolded proteins responsible for neurodegenerative diseases. Autophagy 6, 958–960. 10.4161/auto.6.7.13042 20699640

[B34] Czarny P. Pawlowska E. Bialkowska-Warzecha J. Kaarniranta K. Blasiak J. (2015). Autophagy in DNA damage response. Int. J. Mol. Sci. 16, 2641–2662. 10.3390/ijms16022641 25625517 PMC4346856

[B35] Daigle J. G. Krishnamurthy K. Ramesh N. Casci I. Monaghan J. McAvoy K.. (2016). Pur-alpha regulates cytoplasmic stress granule dynamics and ameliorates FUS toxicity. Acta Neuropathol. 131, 605–620. 10.1007/s00401-015-1530-0 26728149 PMC4791193

[B36] D’Amico E. Factor-Litvak P. Santella R. M. Mitsumoto H. (2013). Clinical perspective on oxidative stress in sporadic amyotrophic lateral sclerosis. Free Radic. Biol. Med. 65, 509–527. 10.1016/j.freeradbiomed.2013.06.029 23797033 PMC3859834

[B37] Damme M. Suntio T. Saftig P. Eskelinen E. L. (2015). Autophagy in neuronal cells: general principles and physiological and pathological functions. Acta Neuropathol. 129, 337–362. 10.1007/s00401-014-1361-4 25367385

[B38] De duve C. Pressman B. C. Gianetto R. Wattiaux R. Appelmans F. (1955). Tissue fractionation studies. 6. Intracellular distribution patterns of enzymes in rat-liver tissue. Biochem. J. 60, 604–617. 10.1042/bj0600604 13249955 PMC1216159

[B39] DeJesus-Hernandez M. Mackenzie I. R. Boeve B. F. Boxer A. L. Baker M. Rutherford N. J.. (2011). Expanded GGGGCC hexanucleotide repeat in noncoding region of *C9ORF72* causes chromosome 9p-linked FTD and ALS. Neuron 72, 245–256. 10.1016/j.neuron.2011.09.011 21944778 PMC3202986

[B41] Deng H. X. Bigio E. H. Zhai H. Fecto F. Ajroud K. Shi Y.. (2011a). Differential involvement of optineurin in amyotrophic lateral sclerosis with or without SOD1 mutations. Arch. Neurol. 68, 1057–1061. 10.1001/archneurol.2011.178 21825243 PMC3357952

[B42] Deng H. X. Chen W. Hong S. T. Boycott K. M. Gorrie G. H. Siddique N.. (2011b). Mutations in UBQLN2 cause dominant X-linked juvenile and adult-onset alS and ALS/dementia. Nature 477, 211–215. 10.1038/nature10353 21857683 PMC3169705

[B40] Deng H. Gao K. Jankovic J. (2014). The role of FUS gene variants in neurodegenerative diseases. Nat. Rev. Neurol. 10, 337–348. 10.1038/nrneurol.2014.78 24840975

[B43] Deng H. X. Zhai H. Bigio E. H. Yan J. Fecto F. Ajroud K.. (2010). FUS-Immunoreactive inclusions are a common feature in sporadic and Non-SOD1 familial amyotrophic lateral sclerosis. Ann. Neurol. 67, 739–748. 10.1002/ana.22051 20517935 PMC4376270

[B44] Díaz-Troya S. Pérez-Pérez M. E. Florencio F. J. Crespo J. L. (2008). The role of TOR in autophagy regulation from yeast to plants and mammals. Autophagy 4, 851–865. 10.4161/auto.6555 18670193

[B45] Doi H. Okamura K. Bauer P. O. Furukawa Y. Shimizu H. Kurosawa M.. (2008). RNA-Binding protein TLS is a major nuclear aggregate-interacting protein in huntingtin exon 1 with expanded polyglutamine-expressing cells. J. Biol. Chem. 283, 6489–6500. 10.1074/jbc.m705306200 18167354

[B46] Dunn W. A. (1994). Autophagy and related mechanisms of lysosome-mediated protein degradation. Trends Cell Biol. 4, 139–143. 10.1016/0962-8924(94)90069-8 14731737

[B47] Duran A. Amanchy R. Linares J. F. Joshi J. Abu-Baker S. Porollo A. (2011). p62 is a key regulator of nutrient sensing in the mTORC1 pathway. Mol. Cell 44, 134–146. 10.1016/j.molcel.2011.06.038 21981924 PMC3190169

[B48] Eliopoulos A. G. Havaki S. Gorgoulis V. G. (2016). DNA damage response and autophagy: a meaningful partnership. Front. Genet. 7:204. 10.3389/fgene.2016.00204 27917193 PMC5116470

[B49] Eskelinen E. L. Saftig P. (2009). Autophagy: a lysosomal degradation pathway with a central role in health and disease. Biochim. Biophys. Acta 1793, 664–673. 10.1016/j.bbamcr.2008.07.014 18706940

[B50] Fan A. C. Leung A. K. (2016). RNA granules and diseases: a case study of stress granules in ALS and FTLD. Adv. Exp. Med. Biol. 907, 263–296. 10.1007/978-3-319-29073-7_11 27256390 PMC5247449

[B51] Farg M. A. Soo K. Y. Walker A. K. Pham H. Orian J. Horne M. K.. (2012). Mutant FUS induces endoplasmic reticulum stress in amyotrophic lateral sclerosis and interacts with protein disulfide-isomerase. Neurobiol. Aging 33, 2855–2868. 10.1016/j.neurobiolaging.2012.02.009 22459602

[B52] Farg M. A. Sundaramoorthy V. Sultana J. M. Yang S. Atkinson R. A. Levina V.. (2014). C9ORF72, implicated in amytrophic lateral sclerosis and frontotemporal dementia, regulates endosomal trafficking. Hum. Mol. Genet. 23, 3579–3595. 10.1093/hmg/ddu068 24549040 PMC4049310

[B53] Finelli M. J. Liu K. X. Wu Y. Oliver P. L. Davies K. E. (2015). Oxr1 improves pathogenic cellular features of ALS-associated FUS and TDP-43 mutations. Hum. Mol. Genet. 24, 3529–3544. 10.1093/hmg/ddv104 25792726 PMC4498158

[B54] Fornai F. Longone P. Cafaro L. Kastsiuchenka O. Ferrucci M. Manca M. L.. (2008). Lithium delays progression of amyotrophic lateral sclerosis. Proc. Natl. Acad. Sci. U S A 105, 2052–2057. 10.1073/pnas.0708022105 18250315 PMC2538879

[B55] Forsberg K. Jonsson P. A. Andersen P. M. Bergemalm D. Graffmo K. S. Hultdin M.. (2010). Novel antibodies reveal inclusions containing non-native SOD1 in sporadic ALS patients. PLoS One 5:e11552. 10.1371/journal.pone.0011552 20644736 PMC2904380

[B56] Gan L. Johnson J. A. (2014). Oxidative damage and the Nrf2-ARE pathway in neurodegenerative diseases. Biochim. Biophys. Acta 1842, 1208–1218. 10.1016/j.bbadis.2013.12.011 24382478

[B57] Gill A. Kidd J. Vieira F. Thompson K. Perrin S. (2009). No benefit from chronic lithium dosing in a sibling-matched, gender balanced, investigator-blinded trial using a standard mouse model of familial ALS. PLoS One 4:e6489. 10.1371/journal.pone.0006489 19649300 PMC2714460

[B58] Giordana M. T. Piccinini M. Grifoni S. De Marco G. Vercellino M. Magistrello M.. (2010). TDP-43 redistribution is an early event in sporadic amyotrophic lateral sclerosis. Brain Pathol. 20, 351–360. 10.1111/j.1750-3639.2009.00284.x 19338576 PMC8094784

[B59] Glick D. Barth S. Macleod K. F. (2010). Autophagy: cellular and molecular mechanisms. J. Pathol. 221, 3–12. 10.1002/path.2697 20225336 PMC2990190

[B60] Goode A. Rea S. Sultana M. Shaw B. Searle M. S. Layfield R. (2016). ALS-FTLD associated mutations of SQSTM1 impact on Keap1-Nrf2 signalling. Mol. Cell. Neurosci. 76, 52–58. 10.1016/j.mcn.2016.08.004 27554286 PMC5062946

[B61] Guerriero C. J. Brodsky J. L. (2012). The delicate balance between secreted protein folding and endoplasmic reticulum-associated degradation in human physiology. Physiolog. Rev. 92, 537–576. 10.1152/physrev.00027.2011 22535891 PMC4162396

[B62] Guo X. Sun X. Hu D. Wang Y. J. Fujioka H. Vyas R.. (2016). VCP recruitment to mitochondria causes mitophagy impairment and neurodegeneration in models of Huntington’s disease. Nat. Com. 7:12646. 10.1038/ncomms12646 27561680 PMC5007466

[B63] Haack T. B. Ignatius E. Calvo-Garrido J. Iuso A. Isohanni P. Maffezzini C.. (2016). Absence of the autophagy adaptor SQSTM1/p62 causes childhood-onset neurodegeneration with ataxia, dystonia and gaze palsy. Am. J. Hum. Genet. 99, 735–743. 10.1016/j.ajhg.2016.06.026 27545679 PMC5010644

[B64] Haeusler A. R. Donnelly C. J. Rothstein J. D. (2016). The expanding biology of the Corf72 nucleotide repeat expansion in neurodegenerative disease. Nat. Rev. Neurosci. 17, 383–395. 10.1038/nrn.2016.38 27150398 PMC7376590

[B65] Hara T. Nakamura K. Matsui M. Yamamoto A. Nakahara Y. Suzuki-Migishima R.. (2006). Suppression of basal autophagy in neural cells causes neurodegenerative disease in mice. Nature 441, 885–889. 10.1038/nature04724 16625204

[B66] Higelin J. Demestre M. Putz S. Delling J. P. Jacob C. Lutz A. K.. (2016). FUS mislocalization and vulnerability to DNA damage in ALS patients derived hiPSCs and aging motoneurons. Front. Cell. Neurosci. 10:290. 10.3389/fncel.2016.00290 28082870 PMC5183648

[B67] Hjerpe R. Bett J. S. Keuss M. J. Solovyova A. McWilliams T. G. Johnson C.. (2016). UBQLN2 mediates autophagy-independent protein aggregate clearance by the proteasome. Cell 166, 935–949. 10.1016/j.cell.2016.07.001 27477512 PMC5003816

[B68] Hortobágyi T. Troakes C. Nishimura A. L. Vance C. van Swieten J. C. Seelaar H.. (2011). Optineurin inclusions occur in a minority of TDP-43 positive ALS and FTLD-TDP cases and are rarely observed in other neurodegenerative disorders. Acta Neuropathol. 121, 519–527. 10.1007/s00401-011-0813-3 21360076

[B69] Hsueh K. W. Chiou T. W. Chiang S. F. Yamashita T. Abe K. Borlongan C. V.. (2016). Autophagic down-Regulation in motor neurons remarkably prolongs the survival of ALS mice. Neuropharmacology 108, 152–160. 10.1016/j.neuropharm.2016.03.035 27059126

[B70] Huang B. Wu Q. Zhou H. Huang C. Xia X. G. (2016). Increased Ubqln2 expression causes neuron death in transgenic rats. J. Neurochem. 139, 285–293. 10.1111/jnc.13748 27456931 PMC5117623

[B71] Huang C. Zhou H. Tong J. Chen H. Liu Y. J. Wang D.. (2011). FUS transgenic rats develop the phenotypes of amyotrophic lateral sclerosis and frontotemporal lobar degeneration. PLoS Genet. 7:e1002011. 10.1371/journal.pgen.1002011 21408206 PMC3048370

[B72] Hurley J. H. Young L. N. (2017). Mechanisms of autophagy initiation. Annu. Rev. Biochem. 86, 225–244. 10.1146/annurev-biochem-061516-044820 28301741 PMC5604869

[B73] Ikenaka K. Kawai K. Katsuno M. Huang Z. Jiang Y. M. Iguchi Y.. (2013). Dnc-1/dynactin 1 knockdown disrupts transport of autophagosomes and induces motor neuron degeneration. PLoS One 8:e54511. 10.1371/journal.pone.0054511 23408943 PMC3567092

[B74] Ilieva E. V. Ayala V. Jové M. Dalfó E. Cacabelos D. Povedano M.. (2007). Oxidative and endoplasmic reticulum stress interplay in sporadic amyotrophic lateral sclerosis. Brain 130, 3111–3123. 10.1093/brain/awm190 17716997

[B75] Ittner L. M. Halliday G. M. Kril J. J. Götz J. Hodges J. R. Kiernan M. C. (2015). FTD and ALS—translating mouse studies into clinical trials. Nat. Rev. Neurol. 11, 360–366. 10.1038/nrneurol.2015.65 25939274

[B76] Jung C. H. Ro S. H. Cao J. Otto N. M. Kim D. H. (2010). mTOR regulation of autophagy. FEBS Lett. 584, 1287–1295. 10.1016/j.febslet.2010.01.017 20083114 PMC2846630

[B77] Kabuta T. Suzuki Y. Wada K. (2006). Degradation of amyotrophic lateral sclerosis-linked mutant Cu,Zn-Superoxide dismutase proteins by macroautophagy and the proteasome. J. Biol. Chem. 281, 30524–30533. 10.1074/jbc.M603337200 16920710

[B78] Kalvakolanu D. V. Gade P. (2012). IFNG and autophagy: a critical role for the ER-stress mediator ATF6 in controlling bacterial infections. Autophagy 8, 1673–1674. 10.4161/auto.21403 22874566 PMC3494595

[B79] Kanno T. Tanaka K. Yanagisawa Y. Yasutake K. Hadano S. Yoshii F.. (2012). A novel small molecule, N-(4–(2-pyridyl)(1,3-Thiazol-2-Yl))-2–(2,4,6-Trimethylphenoxy) acetamide, selectively protects against oxidative stress-induced cell death by activating the Nrf2-ARE pathway: therapeutic implications for ALS. Free Radic. Biol. Med. 53, 2028–2042. 10.1016/j.freeradbiomed.2012.09.010 23000247

[B80] Kato S. Takikawa M. Nakashima K. Hirano A. Cleveland D. W. Kusaka H.. (2000). New consensus research on neuropathological aspects of familial amyotrophic lateral sclerosis with superoxide dismutase 1 (SOD1) gene mutations: inclusions containing SOD1 in neurons and astrocytes. Amyotroph. Lateral Scler. Other Motor Neuron Disord. 1, 163–184. 10.1080/14660820050515160 11464950

[B81] Kesidou E. Lagoudaki R. Touloumi O. Poulatsidou K. N. Simeonidou C. (2013). Autophagy and neurodegenerative disorders. Neural Regen. Res. 8, 2275–2283. 10.3969/j.issn.1673-5374.2013.24.007 25206537 PMC4146038

[B82] Klionsky D. J. Eskelinen E. L. Deretic V. (2014). Autophagosomes, phagosomes, autolysosomes, phagolysosomes, autophagolysosomes… wait, I’m confused. Autophagy 10, 549–551. 10.4161/auto.28448 24657946 PMC4091142

[B83] Komatsu M. Waguri S. Chiba T. Murata S. Iwata J. Tanida I.. (2006). Loss of autophagy in the central nervous system causes neurodegeneration in mice. Nature 441, 880–884. 10.1038/nature04723 16625205

[B84] Kuo S. Y. Castoreno A. B. Aldrich L. N. Lassen K. G. Goel G. Dancík V.. (2015). Small-molecule enhancers of autophagy modulate cellular disease phenotypes suggested by human genetics. Proc. Natl. Acad. Sci. U S A 112, E4281–E4287. 10.1073/pnas.1512289112 26195741 PMC4534235

[B85] Kwiatkowski T. J. Jr. Bosco D. A. Leclerc A. L. Tamrazian E. Vanderburg C. R. Russ C.. (2009). Mutations in the FUS/TLS gene on chromosome 16 cause familial amyotrophic lateral sclerosis. Science 323, 1205–1208. 10.1126/science.1166066 19251627

[B86] Lagier-Tourenne C. Polymenidou M. Cleveland D. W. (2010). TDP-43 and FUS/TLS: emerging roles in RNA processing and neurodegeneration. Hum. Mol. Genet. 19, R46–R64. 10.1093/hmg/ddq137 20400460 PMC3167692

[B87] Laird F. M. Farah M. H. Ackerley S. Hoke A. Maragakis N. Rothstein J. D.. (2008). Motor neuron disease occurring in a mutant dynactin mouse model is characterized by defects in vesicular trafficking. J. Neurosci. 28, 1997–2005. 10.1523/JNEUROSCI.4231-07.2008 18305234 PMC6671836

[B88] Lattante S. de Calbiac H. Le Ber I. Brice A. Ciura S. Kabashi E. (2015). Sqstm1 knock-down causes a locomotor phenotype ameliorated by rapamycin in a Zebrafish model of ALS/FTLD. Hum. Mol. Genet. 24, 1682–1690. 10.1093/hmg/ddu580 25410659

[B89] Lazarou M. Sliter D. A. Kane L. A. Sarraf S. A. Wang C. Burman J. L.. (2015). The ubiquitin kinase PINK1 recruits autophagy receptors to induce mitophagy. Nature 524, 309–314. 10.1038/nature14893 26266977 PMC5018156

[B90] Lee J. Giordano S. Zhang J. (2012). Autophagy, mitochondria and oxidative stress: cross-talk and redox signalling. Biochem. J. 441, 523–540. 10.1042/bj20111451 22187934 PMC3258656

[B92] Li Y. Guo Y. Wang X. Yu X. Duan W. Hong K.. (2015). Trehalose decreases mutant SOD1 expression and alleviates motor deficiency in early but not end-stage amyotrophic lateral sclerosis in a SOD1–G93A mouse model. Neuroscience 298, 12–25. 10.1016/j.neuroscience.2015.03.061 25841320

[B91] Li F. Xie X. Wang Y. Liu J. Cheng X. Guo Y.. (2016). Structural insights into the interaction and disease mechanism of neurodegenerative disease-associated optineurin and TBK1 proteins. Nat. Commun. 7:12708. 10.1038/ncomms12708 27620379 PMC5027247

[B93] Longatti A. Tooze S. A. (2012). Recycling endosomes contribute to autophagosome formation. Autophagy 8, 1682–1683. 10.4161/auto.21486 22874560 PMC3494599

[B94] Lopez-Gonzalez R. Lu Y. Gendron T. F. Karydas A. Tran H. Yang D.. (2016). Poly(GR) in *C9ORF72*-related ALS/FTD compromises mitochondrial function and increases oxidative stress and DNA damage in iPSC-derived motor neurons. Neuron 92, 383–391. 10.1016/j.neuron.2016.09.015 27720481 PMC5111366

[B95] Machado-Vieira R. Manji H. K. Zarate C. A. Jr. (2009). The role of lithium in the treatment of bipolar disorder: convergent evidence for neurotrophic effects as a unifying hypothesis. Bipolar Disord. 11, 92–109. 10.1111/j.1399-5618.2009.00714.x 19538689 PMC2800957

[B96] Mackenzie I. R. Ansorge O. Strong M. Bilbao J. Zinman L. Ang L. C.. (2011). Pathological heterogeneity in amyotrophic lateral sclerosis with FUS mutations: two distinct patterns correlating with disease severity and mutation. Acta Neuropathol. 122, 87–98. 10.1007/s00401-011-0838-7 21604077 PMC3319073

[B97] Mackenzie I. R. Arzberger T. Kremmer E. Troost D. Lorenzl S. Mori K.. (2013). Dipeptide repeat protein pathology in *C9ORF72* mutation cases: clinico-pathological correlations. Acta Neuropathol. 126, 859–879. 10.1007/s00401-013-1181-y 24096617

[B98] Mackenzie I. R. Bigio E. H. Ince P. G. Geser F. Neumann M. Cairns N. J.. (2007). Pathological TDP-43 distinguishes sporadic amyotrophic lateral sclerosis from amyotrophic lateral sclerosis with SOD1 mutations. Ann. Neurol. 61, 427–434. 10.1002/ana.21147 17469116

[B99] Mackenzie I. R. Frick P. Neumann M. (2014). The neuropathology associated with repeat expansions in the *C9ORF72* gene. Acta Neuropathol. 127, 347–357. 10.1007/s00401-013-1232-4 24356984

[B100] Maday S. (2016). Mechanisms of neuronal homeostasis: autophagy in the axon. Brain Res. 1649, 143–150. 10.1016/j.brainres.2016.03.047 27038755 PMC5045311

[B101] Majcher V. Goode A. James V. Layfield R. (2015). Autophagy receptor defects and ALS-FTLD. Mol. Cell. Neurosci. 66, 43–52. 10.1016/j.mcn.2015.01.002 25683489

[B102] Majounie E. Renton A. E. Mok K. Dopper E. G. Waite A. Rollinson S.. (2012). Frequency of the *C9orf72* hexanucleotide repeat expansion in patients with amyotrophic lateral sclerosis and frontotemporal dementia: a cross-sectional study. Lancet Neurol. 11, 323–330. 10.1016/S1474-4422(12)70043-1 22406228 PMC3322422

[B103] Maruyama H. Morino H. Ito H. Izumi Y. Kato H. Watanabe Y.. (2010). Mutations of optineurin in amyotrophic lateral sclerosis. Nature 465, 223–226. 10.1038/nature08971 20428114

[B104] Matus S. Lopez E. Valenzuela V. Nassif M. Hetz C. (2013). Functional contribution of the transcription factor ATF4 to the pathogenesis of amyotrophic lateral sclerosis. PLoS One 8:e66672. 10.1371/journal.pone.0066672 23874395 PMC3715499

[B105] Menzies F. M. Fleming A. Rubinsztein D. C. (2015). Compromised autophagy and neurodegenerative diseases. Nat. Rev. Neurosci. 16, 345–357. 10.1038/nrn3961 25991442

[B106] Millecamps S. Salachas F. Cazeneuve C. Gordon P. Bricka B. Camuzat A.. (2010). SOD1, ANG, VAPB, TARDBP and FUS mutations in familial amyotrophic lateral sclerosis: genotype-phenotype correlations. J. Med. Genet. 47, 554–560. 10.1136/jmg.2010.077180 20577002

[B107] Moore A. S. Holzbaur E. L. (2016). Dynamic recruitment and activation of ALS-associated TBK1 with its target optineurin are required for efficient mitophagy. Proc. Natl. Acad. Sci. U S A 113, E3349–E3358. 10.1073/pnas.1523810113 27247382 PMC4914160

[B108] Mori K. Weng S. M. Arzberger T. May S. Rentzsch K. Kremmer E.. (2013). The *C9orf72* GGGGCC repeat is translated into aggregating dipeptide-repeat proteins in FTLD/ALS. Science 339, 1335–1338. 10.1126/science.1232927 23393093

[B109] Münch C. Sedlmeier R. Meyer T. Homberg V. Sperfeld A. D. Kurt A.. (2004). Point mutations of the p150 subunit of dynactin (DCTN1) gene in ALS. Neurology 63, 724–726. 10.1212/01.wnl.0000134608.83927.b1 15326253

[B110] Muyderman H. Chen T. (2014). Mitochondrial dysfunction in amyotrophic lateral sclerosis—a valid pharmacological target? Br. J. Pharmacol. 171, 2191–2205. 10.1111/bph.12476 24148000 PMC3976630

[B111] Nah J. Yuan J. Jung Y. K. (2015). Autophagy in neurodegenerative diseases: from mechanism to therapeutic approach. Mol. Cells 38, 381–389. 10.14348/molcells.2015.0034 25896254 PMC4443278

[B112] Nassif M. Valenzuela V. Rojas-Rivera D. Vidal R. Matus S. Castillo K.. (2014). Pathogenic role of BECN1/Beclin 1 in the development of amyotrophic lateral sclerosis. Autophagy 10, 1256–1271. 10.4161/auto.28784 24905722 PMC4203551

[B113] Neumann M. Sampathu D. M. Kwong L. K. Truax A. C. Micsenyi M. C. Chou T. T.. (2006). Ubiquitinated TDP-43 in frontotemporal lobar degeneration and amyotrophic lateral sclerosis. Science 314, 130–133. 10.1126/science.1134108 17023659

[B114] Ohsumi Y. (2014). Historical landmarks of autophagy research. Cell Res. 24, 9–23. 10.1038/cr.2013.169 24366340 PMC3879711

[B115] Osaka M. Ito D. Suzuki N. (2016). Disturbance of proteasomal and autophagic protein degradation pathways by amyotrophic lateral sclerosis-linked mutations in ubiquilin 2. Biochem. Biophys. Res. Commun. 472, 324–331. 10.1016/j.bbrc.2016.02.107 26944018

[B116] Osaka M. Ito D. Yagi T. Nihei Y. Suzuki N. (2015). Evidence of a link between ubiquilin 2 and optineurin in amyotrophic lateral sclerosis. Hum. Mol. Genet. 24, 1617–1629. 10.1093/hmg/ddu575 25398946

[B117] Pasinelli P. Brown R. H. (2006). Molecular biology of amyotrophic lateral sclerosis: insights from genetics. Nat. Rev. Neurosci. 7, 710–723. 10.1038/nrn1971 16924260

[B118] Patel A. Lee H. O. Jawerth L. Maharana S. Jahnel M. Hein M. Y.. (2015). A liquid-to-solid phase transition of the ALS protein FUS accelerated by disease mutation. Cell 162, 1066–1077. 10.1016/j.cell.2015.07.047 26317470

[B119] Pizzasegola C. Caron I. Daleno C. Ronchi A. Minoia C. Carrì M. T. (2009). Treatment with lithium carbonate does not improve disease progression in two different strains of SOD1 mutant mice. Amyotroph. Lateral Scler. 10, 221–228. 10.1080/17482960902803440 19308767

[B120] Qin H. Lim L. Z. Wei Y. Song J. (2014). TDP-43 N terminus encodes a novel ubiquitin-like fold and its unfolded form in equilibrium that can be shifted by binding to ssDNA. Proc. Natl. Acad. Sci. U S A 111, 18619–18624. 10.1073/pnas.1413994112 25503365 PMC4284588

[B121] Qiu H. Lee S. Shang Y. Wang W. Y. Au K. F. Kamiya S.. (2014). ALS-associated mutation FUS-R521C causes DNA damage and RNA splicing defects. J. Clin. Invest. 124, 981–999. 10.1172/JCI72723 24509083 PMC3938263

[B122] Rashid H. O. Yadav R. K. Kim H. R. Chae H. J. (2015). ER stress: autophagy induction, inhibition and selection. Autophagy 11, 1956–1977. 10.1080/15548627.2015.1091141 26389781 PMC4824587

[B123] Ravikumar B. Futter M. Jahreiss L. Korolchuk V. I. Lichtenberg M. Luo S.. (2009). Mammalian macroautophagy at a glance. J. Cell Sci. 122, 1707–1711. 10.1242/jcs.031773 19461070 PMC2684830

[B124] Rea S. L. Walsh J. P. Layfield R. Ratajczak T. Xu J. (2013). New insights into the role of sequestosome 1/p62 mutant proteins in the pathogenesis of paget’s disease of bone. Endocr. Rev. 34, 501–524. 10.1210/er.2012-1034 23612225

[B125] Reggiori F. Klionsky D. J. (2002). Autophagy in the eukaryotic cell. Eukaryot. Cell 1, 11–21. 10.1128/ec.01.1.11-21.2002 12455967 PMC118053

[B126] Renton A. E. Chiò A. Traynor B. J. (2014). State of play in amyotrophic lateral sclerosis genetics. Nat. Neurosci. 17, 17–23. 10.1038/nn.3584 24369373 PMC4544832

[B127] Renton A. E. Majounie E. Waite A. Simón-Sánchez J. Rollinson S. Gibbs J. R.. (2011). A hexanucleotide repeat expansion in *C9ORF72* is the cause of chromosome 9p21-linked ALS-FTD. Neuron 72, 257–268. 10.1016/j.neuron.2011.09.010 21944779 PMC3200438

[B128] Rosen D. R. Siddique T. Patterson D. Figlewicz D. A. Sapp P. Hentati A.. (1993). Mutations in Cu/Zn superoxide dismutase gene are associated with familial amyotrophic lateral sclerosis. Nature 362, 59–62. 10.1038/362059a0 8446170

[B129] Ross C. A. Poirier M. A. (2004). Protein aggregation and neurodegenerative disease. Nat. Med. 10, S10–S17. 10.1038/nm1066 15272267

[B130] Rothenberg C. Srinivasan D. Mah L. Kaushik S. Peterhoff C. M. Ugolino J.. (2010). Ubiquilin functions in autophagy and is degraded by chaperone-mediated autophagy. Hum. Mol. Genet. 19, 3219–3232. 10.1093/hmg/ddq231 20529957 PMC2908472

[B131] Rowland L. P. Shneider N. A. (2001). Amyotrophic lateral sclerosis. N Engl J. Med. 344, 1688–1700. 10.1056/NEJM200105313442207 11386269

[B132] Rubinsztein D. C. Shpilka T. Elazar Z. (2012). Mechanisms of autophagosome biogenesis. Curr. Biol. 22, R29–R34. 10.1016/j.cub.2011.11.034 22240478

[B133] Ryu H. H. Jun M. H. Min K. J. Jang D. J. Lee Y. S. Kim H. K.. (2014). Autophagy regulates amyotrophic lateral sclerosis-linked fused in sarcoma-positive stress granules in neurons. Neurobiol. Aging 35, 2822–2831. 10.1016/j.neurobiolaging.2014.07.026 25216585

[B134] Sama R. R. Ward C. L. Bosco D. A. (2014). Functions of FUS/TLS from DNA repair to stress response: implications for ALS. ASN Neuro 6:1759091414544472. 10.1177/1759091414544472 25289647 PMC4189536

[B135] Sarkar S. Rubinsztein D. C. (2008). Small molecule enhancers of autophagy for neurodegenerative diseases. Mol. Biosyst. 4, 895–901. 10.1039/b804606a 18704227

[B136] Sasaki S. (2011). Autophagy in spinal cord motor neurons in sporadic amyotrophic lateral sclerosis. J. Neuropathol. Exp. Neurol. 70, 349–359. 10.1097/nen.0b013e3182160690 21487309

[B137] Scotter E. L. Chen H. J. Shaw C. E. (2015). Erratum to: TDP-43 proteinopathy and ALS: insights into disease mechanisms and therapeutic targets. Neurotherapeutics 12, 515–518. 10.1007/s13311-015-0351-0 25761971 PMC4404465

[B138] Seguin S. J. Morelli F. F. Vinet J. Amore D. De Biasi S. Poletti A.. (2014). Inhibition of autophagy, lysosome and VCP function impairs stress granule assembly. Cell Death Differ. 21, 1838–1851. 10.1038/cdd.2014.103 25034784 PMC4227144

[B139] Sellier C. Campanari M. L. Julie Corbier C. Gaucherot A. Kolb-Cheynel I. Oulad-Abdelghani M.. (2016). Loss of C9ORF72 impairs autophagy and synergizes with polyQ Ataxin-2 to induce motor neuron dysfunction and cell death. EMBO J. 35, 1276–1297. 10.15252/embj.201593350 27103069 PMC4910533

[B140] Shen W.-C. Li H.-Y. Chen G.-C. Chern Y. Tu P. H. (2015). Mutations in the ubiquitin-binding domain of OPTN/optineurin interfere with autophagy-mediated degradation of misfolded proteins by a dominant-negative mechanism. Autophagy 11, 685–700. 10.4161/auto.36098 25484089 PMC4502753

[B141] Shibata N. Asayama K. Hirano A. Kobayashi M. (1996a). Immunohistochemical study on superoxide dismutases in spinal cords from autopsied patients with amyotrophic lateral sclerosis. Dev. Neurosci. 18, 492–498. 10.1159/000111445 8940623

[B143] Shibata N. Hirano A. Kobayashi M. Siddique T. Deng H. X. Hung W. Y.. (1996b). Intense superoxide dismutase-1 immunoreactivity in intracytoplasmic hyaline inclusions of familial amyotrophic lateral sclerosis with posterior column involvement. J. Neuropathol. Exp. Neurol. 55, 481–490. 10.1097/00005072-199604000-00011 8786408

[B142] Shibata N. Hirano A. Kobayashi M. Sasaki S. Kato T. Matsumoto S.. (1994). Cu/Zn superoxide dismutase-like immunoreactivity in lewy body-like inclusions of sporadic amyotrophic lateral sclerosis. Neurosci. Lett. 179, 149–152. 10.1016/0304-3940(94)90956-3 7845611

[B144] Shintani T. Klionsky D. J. (2004). Autophagy in health and disease: a double-edged sword. Science 306, 990–995. 10.1126/science.1099993 15528435 PMC1705980

[B145] Soo K. Y. Sultana J. King A. E. Atkinson R. Warraich S. T. Sundaramoorthy V.. (2015). ALS-associated mutant FUS inhibits macroautophagy which is restored by overexpression of Rab1. Cell Death Discov. 1:15030. 10.1038/cddiscovery.2015.30 27551461 PMC4979432

[B146] Sreedharan J. Blair I. P. Tripathi V. B. Hu X. Vance C. Rogelj B.. (2008). TDP-43 mutations in familial and sporadic amyotrophic lateral sclerosis. Science 319, 1668–1672. 10.1126/science.1154584 18309045 PMC7116650

[B147] Sullivan P. M. Zhou X. Robins A. M. Paushter D. H. Kim D. Smolka M. B.. (2016). The ALS/FTLD associated protein C9orf72 associates with SMCR8 and WDR41 to regulate the autophagy-lysosome pathway. Acta Neuropathol. Commun. 4:51. 10.1186/s40478-016-0324-5 27193190 PMC4870812

[B148] Szatmári Z. Kis V. Lippai M. Hegedus K. Faragó T. Lorincz P.. (2014). Rab11 facilitates cross-talk between autophagy and endosomal pathway through regulation of hook localization. Mol. Biol. Cell 25, 522–531. 10.1091/mbc.e13-10-0574 24356450 PMC3923643

[B149] Tan C. F. Eguchi H. Tagawa A. Onodera O. Iwasaki T. Tsujino A.. (2007). TDP-43 immunoreactivity in neuronal inclusions in familial amyotrophic lateral sclerosis with or without sod1 gene mutation. Acta Neuropathol. 113, 535–542. 10.1007/s00401-007-0206-9 17333220

[B150] Tanida I. Ueno T. Kominami E. (2008). “LC3 and autophagy BT—autophagosome and phagosome,” in Methods in Molecular Biology, ed. Deretic V. (Totowa, NJ: Humana Press), 77–88. 10.1007/978-1-59745-157-4_4 18425443

[B151] Teyssou E. Takeda T. Lebon V. Boillée S. Doukouré B. Bataillon G.. (2013). Mutations in SQSTM1 encoding p62 in amyotrophic lateral sclerosis: genetics and neuropathology. Acta Neuropathol. 125, 511–522. 10.1007/s00401-013-1090-0 23417734

[B152] Tokuda E. Brännström T. Andersen P. M. Marklund S. L. (2016). Low autophagy capacity implicated in motor system vulnerability to mutant superoxide dismutase. Acta Neuropathol. Commun. 4:6. 10.1186/s40478-016-0274-y 26810478 PMC4727314

[B153] Traynor B. J. Alexander M. Corr B. Frost E. Hardiman O. (2003). An outcome study of riluzole in amyotrophic lateral sclerosis–a population-based study in Ireland, 1996–2000. J. Neurol. 250, 473–479. 10.1007/s00415-003-1026-z 12700914

[B154] Turner M. R. Hardiman O. Benatar M. Brooks B. R. Chio A. de Carvalho M.. (2013). Controversies and priorities in amyotrophic lateral sclerosis. Lancet Neurol. 12, 310–322. 10.1016/S1474-4422(13)70036-X 23415570 PMC4565161

[B155] Ugolino J. Ji Y. J. Conchina K. Chu J. Nirujogi R. S. Pandey A.. (2016). Loss of C9orf72 enhances autophagic activity via deregulated mTOR and TFEB signaling. PLoS Genet. 12:e1006443. 10.1371/journal.pgen.1006443 27875531 PMC5119725

[B156] Ugras S. E. Shorter J. (2012). RNA-binding proteins in amyotrophic lateral sclerosis and neurodegeneration. Neurol. Res. Int. 2012:432780. 10.1155/2012/432780 22919483 PMC3423945

[B157] Van Deerlin V. M. Leverenz J. B. Bekris L. M. Bird T. D. Yuan W. Elman L. B.. (2008). TARDBP mutations in amyotrophic lateral sclerosis with TDP-43 neuropathology: a genetic and histopathological analysis. Lancet Neurol. 7, 409–416. 10.1016/S1474-4422(08)70071-1 18396105 PMC3546119

[B158] Vance C. Rogelj B. Hortobágyi T. De Vos K. J. Nishimura A. L. Sreedharan J.. (2009). Mutations in FUS, an RNA processing protein, cause familial amyotrophic lateral sclerosis type 6. Science 323, 1208–1211. 10.1126/science.1165942 19251628 PMC4516382

[B159] Vlug A. S. Teuling E. Haasdijk E. D. French P. Hoogenraad C. C. Jaarsma D. (2005). ATF3 expression precedes death of spinal motoneurons in amyotrophic lateral sclerosis-SOD1 transgenic mice and correlates with c-Jun phosphorylation, CHOP expression, somato-dendritic ubiquitination and golgi fragmentation. Eur. J. Neurosci. 22, 1881–1894. 10.1111/j.1460-9568.2005.04389.x 16262628

[B160] Walker A. K. Soo K. Y. Sundaramoorthy V. Parakh S. Ma Y. Farg M. A.. (2013). ALS-associated TDP-43 induces endoplasmic reticulum stress, which drives cytoplasmic TDP-43 accumulation and stress granule formation. PLoS One 8:e81170. 10.1371/journal.pone.0081170 24312274 PMC3843686

[B165] Wang X. Fan H. Ying Z. Li B. Wang H. Wang G. (2010). Degradation of TDP-43 and its pathogenic form by autophagy and the ubiquitin-proteasome system. Neurosci. Lett. 469, 112–116. 10.1016/j.neulet.2009.11.055 19944744

[B163] Wang T. Ming Z. Xiaochun W. Hong W. (2011). Rab7: role of its protein interaction cascades in endo-lysosomal traffic. Cell. Signal. 23, 516–521. 10.1016/j.cellsig.2010.09.012 20851765

[B164] Wang W.-Y. Pan L. Su S. C. Quinn E. J. Sasaki M. Jimenez J. C.. (2013). Interaction of FUS and HDAC1 regulates DNA damage response and repair in neurons. Nat. Neurosci. 16, 1383–1391. 10.1038/nn.3514 24036913 PMC5564396

[B161] Wang I.-F. Tsai K.-J. Shen C.-K. (2013). Autophagy activation ameliorates neuronal pathogenesis of FTLD-U mice: a new light for treatment of TARDBP/TDP-43 proteinopathies. Autophagy 9, 239–240. 10.4161/auto.22526 23108236 PMC3552888

[B162] Wang J. Xu G. Borchelt D. R. (2006). Mapping superoxide dismutase 1 Domains of non-native interaction: roles of intra- and intermolecular disulfide bonding in aggregation. J. Neurochem. 96, 1277–1288. 10.1111/j.1471-4159.2005.03642.x 16441516 PMC3989867

[B166] Watanabe M. Dykes-Hoberg M. Culotta V. C. Price D. L. Wong P. C. Rothstein J. D. (2001). Histological evidence of protein aggregation in mutant SOD1 transgenic mice and in amyotrophic lateral sclerosis neural tissues. Neurobiol. Dis. 8, 933–941. 10.1006/nbdi.2001.0443 11741389

[B167] Webster C. P. Smith E. F. Bauer C. S. Moller A. Hautbergue G. M. Ferraiuolo L.. (2016). The C9orf72 protein interacts with Rab1a and the ULK1 complex to regulate initiation of autophagy. EMBO J. 35, 1656–1676. 10.15252/embj.201694401 27334615 PMC4969571

[B168] Williams K. L. Warraich S. T. Yang S. Solski J. A. Fernando R. Rouleau G. A.. (2012). UBQLN2/ubiquilin 2 mutation and pathology in familial amyotrophic lateral sclerosis. Neurobiol. Aging 33, 2527.e3–2527.e10. 10.1016/j.neurobiolaging.2012.05.008 22717235

[B169] Wong Y. C. Holzbaur E. L. (2014). Optineurin is an autophagy receptor for damaged mitochondria in parkin-mediated mitophagy that is disrupted by an ALS-linked mutation. Proc. Natl. Acad. Sci. U S A 111, E4439–E4448. 10.1073/pnas.1405752111 25294927 PMC4210283

[B170] Wong Y. C. Holzbaur E. L. (2015). Temporal dynamics of PARK2/parkin and OPTN/optineurin recruitment during the mitophagy of damaged mitochondria. Autophagy 11, 422–424. 10.1080/15548627.2015.1009792 25801386 PMC4502688

[B171] Wooten M. W. Hu X. Babu J. R. Seibenhener M. L. Geetha T. Paine M. G.. (2006). Signaling, polyubiquitination, trafficking, and inclusions: sequestosome 1/p62’s role in neurodegenerative disease. J. Biomed. Biotechnol. 2006:62079. 10.1155/jbb/2006/62079 17047309 PMC1559922

[B172] Wu Q. Liu M. Huang C. Liu X. Huang B. Li N.. (2015). Pathogenic Ubqln2 gains toxic properties to induce neuron death. Acta Neuropathol. 129, 417–428. 10.1007/s00401-014-1367-y 25388785 PMC4777328

[B173] Xia Q. Wang H. Hao Z. Fu C. Hu Q. Gao F.. (2016). TDP-43 loss of function increases TFEB activity and blocks autophagosome-lysosome fusion. EMBO J. 35, 121–142. 10.15252/embj.201591998 26702100 PMC4718457

[B174] Xie Y. Zhou B. Lin M. Y. Wang S. Foust K. D. Sheng Z. H. (2015). Endolysosomal deficits augment mitochondria pathology in spinal motor neurons of asymptomatic fALS mice. Neuron 87, 355–370. 10.1016/j.neuron.2015.06.026 26182418 PMC4511489

[B175] Ying H. Yue B. Y. (2012). Cellular and molecular biology of optineurin. Int. Rev. Cell Mol. Biol. 294, 223–258. 10.1016/B978-0-12-394305-7.00005-7 22364875 PMC3673586

[B3] Yoshino H. Kimura A. (2006). Investigation of the therapeutic effects of edaravone, a free radical scavenger, on amyotrophic lateral sclerosis (Phase II study). Amyotroph. Lateral Scler. 7, 247–251. 10.1080/17482960600881870 17127563

[B176] Yung C. Sha D. Li L. Chin L. S. (2016). Parkin protects against misfolded SOD1 toxicity by promoting its aggresome formation and autophagic clearance. Mol. Neurobiol. 53, 6270–6287. 10.1007/s12035-015-9537-z 26563499 PMC4866905

[B177] Zarei S. Carr K. Reiley L. Diaz K. Guerra O. Altamirano P. F.. (2015). A comprehensive review of amyotrophic lateral sclerosis. Surg. Neurol. Int. 6:171. 10.4103/2152-7806.169561 26629397 PMC4653353

[B182] Zhang J. (2013). Autophagy and mitophagy in cellular damage control. Redox Biol. 1, 19–23. 10.1016/j.redox.2012.11.008 23946931 PMC3740586

[B180] Zhang X. Chen S. Song L. Tang Y. Shen Y. Jia L.. (2014). MTOR-independent, autophagic enhancer trehalose prolongs motor neuron survival and ameliorates the autophagic flux defect in a mouse model of amyotrophic lateral sclerosis. Autophagy 10, 588–602. 10.4161/auto.27710 24441414 PMC4091147

[B181] Zhang X. Li L. Chen S. Yang D. Wang Y. Zhang X.. (2011). Rapamycin treatment augments motor neuron degeneration in SOD1^G93A^ mouse model of amyotrophic lateral sclerosis. Autophagy 7, 412–425. 10.4161/auto.7.4.14541 21193837

[B178] Zhang K. Shi P. An T. Wang Q. Wang J. Li Z.. (2013). Food restriction-induced autophagy modulates degradation of mutant SOD1 in an amyotrophic lateral sclerosis mouse model. Brain Res. 1519, 112–119. 10.1016/j.brainres.2013.04.050 23643856

[B179] Zhang L. Yu J. Pan H. Hu P. Hao Y. Cai W.. (2007). Small molecule regulators of autophagy identified by an image-based high-throughput screen. Proc. Natl. Acad. Sci. U S A 104, 19023–19028. 10.1073/pnas.0709695104 18024584 PMC2141901

[B183] Zoghbi H. Y. Orr H. T. (2000). Glutamine repeats and neurodegeneration. Annu. Rev. Neurosci. 23, 217–247. 10.1146/annurev.neuro.23.1.217 10845064

